# Impact of Computer-Mediated Versus Face-to-Face Motivational-Type Interviews on Participants’ Language and Subsequent Cannabis Use: Randomized Controlled Trial

**DOI:** 10.2196/59085

**Published:** 2025-04-25

**Authors:** Karla D Llanes, Jon Amastae, Paul C Amrhein, Nadra Lisha, Katherina Arteaga, Eugene Lopez, Roberto A Moran, Lawrence D Cohn

**Affiliations:** 1 Center for Tobacco Control Research and Education University of California San Francisco San Francisco, CA United States; 2 Department of Language and Linguistics The University of Texas at El Paso El Paso, TX United States; 3 Department of Psychology Montclair University Montclair, NJ United States; 4 Department of Psychology Oklahoma State University Stillwaters, OK United States; 5 Department of Psychology The University of Texas at El Paso El Paso, TX United States

**Keywords:** motivational interviews, computer-mediated, commitment language, change talk, sustain talk, marijuana use, cannabis use, behavior change, randomized study, young adults, marijuana users, substance use

## Abstract

**Background:**

Motivational interviewing (MI) is frequently used to facilitate behavior change. The use of change talk during motivational interviews can predict subsequent behavior change. However, no studies have compared the information obtained from traditional face-to-face motivational interviews and computer-mediated motivational interviews or resulted in the same amount of behavior change.

**Objective:**

This study aimed to investigate if face-to-face motivational-type interviews (MTIs) and computer-mediated MTIs elicit the same amount of “change talk” and behavior change when young adults discuss their ambivalence about using marijuana.

**Methods:**

A total of 150 users, including frequent marijuana users, occasional marijuana users, and non–marijuana users, participated in the study. All participants reported being at least moderately ambivalent about their current level of marijuana use. Participants were randomly assigned to complete a brief MTI using either the standard face-to-face format or a computer-mediated format. Amrhein’s manual for assessing the presence of “change talk” and “sustain talk” was used to code the language produced by respondents in each interview format. A reduction in marijuana use was assessed at a 2-month follow-up.

**Results:**

The word count was significantly higher in face-to-face MTIs compared with computer-mediated MTIs (*P*<.001). After controlling for verbosity, face-to-face MTIs, and computer-mediated MTIs did not differ statistically in the overall amount of change talk (*P*=.47) and sustain talk (*P*=.05). Face-to-face MTIs elicited significantly more reasons for reducing future marijuana use (ie, change talk; *P*=.02) and readiness toward not using marijuana (ie, change talk; *P*=.009), even after controlling for verbosity. However, these differences were not statistically significant after using a conservative Bonferroni correction (*P*<.004). After controlling for marijuana use at Time 1, the relationship between the strength of commitment language at Time 1 and marijuana use at Time 2 was not statistically significant (semipartial correlation *r*=0.03, *P*=.57). The association between Time 1 change talk and Time 2 marijuana use depended on the type of motivational interview that participants experienced: face-to-face MTI versus computer-mediated MTI (B=0.45, *P*=.01). A negative binomial regression with a log link function was used to probe this relationship after controlling for 2 covariates: gender and Time 1 (baseline assessment) marijuana use. Among participants in the face-to-face MTI condition, Time 2 (follow-up) marijuana use decreased as the strength of Time 1 change talk increased, although this finding was not significant (B*=*–0.21, *P*=.08). However, among participants in the computer-mediated MTI condition, Time 2 marijuana use was not significantly related to the strength of Time 1 change talk (B=0.13, *P*=.16).

**Conclusions:**

Computer-mediated MTIs and face-to-face MTIs elicit both change talk and sustain talk, which suggests that motivational interviews could potentially be adapted for delivery via text-based computer platforms. However, further research is needed to enhance the predictive validity of the type of language obtained via computer-delivered MI.

**Trial Registration:**

ClinicalTrials.gov NCT06945471; https://clinicaltrials.gov/study/NCT06945471

## Introduction

### Background

Motivational interviewing (MI) is frequently used to facilitate behavior change. Motivational interviews can be short (eg, 15 min) or long (eg, 12 h distributed across multiple sessions), and both formats are associated with reductions in health-threatening behaviors [1]. More than 200 controlled clinical trials support MI’s efficacy for reducing alcohol use, drug use, and poor dietary choices. Studies also support the use of MI for helping individuals manage a range of medical conditions such as asthma and diabetes [1-3]. A meta-analysis of 119 MI efficacy studies yielded a small but significant effect of MI compared with alternative interventions (*d*=0.22) [1].

More than 40 studies have investigated the feasibility of conducting motivational interviews delivered partially or completely via computers, mobile phones, robots, voice-activated recordings, and related platforms [4,5]. Many of these studies have used text-based motivational interviews that rely on written interactions between the interviewer and interviewee. Such text-based MIs have used a variety of communication modes, including chat boxes [6,7], chatbots [8-10], and avatars [6]. Outcome variables included behavior change (eg, decreasing tobacco use and increasing physical activity), self-reported readiness for behavior change, MI session engagement, and perceived interviewer empathy [6,8]. However, no studies have compared the information obtained from (1) traditional face-to-face (FTF) motivational interviews and (2) technology-assisted MIs. It is not known if FTF MIs and technology-assisted MIs elicit the same number of words and thoughts during the interview process, nor is it known if traditional FTF MIs and technology-assisted MIs elicit the same amount of verbal commitment to changing a target behavior (eg, reducing drug use). Finally, it is not known if FTF MIs and technology-assisted MIs elicit the same amount of change talk, sustain talk, and behavior change. The current research begins to address these gaps in knowledge by comparing the language elicited from participants during FTF motivational-type interviews (MTIs) and text-based (computer-mediated) MTIs.

### Computer-Mediated Communications and FTF Communications

Computer-mediated communications (CMCs) are either synchronized or asynchronized. Asynchronized CMCs have delayed responses (eg, email). In contrast, synchronized CMCs (eg, online chat rooms) closely resemble FTF communication by providing participants with immediate responses that facilitate interpersonal exchanges. Many studies have investigated if CMCs elicit greater self-disclosure than FTF communications [11,12]. A meta-analysis of 31 studies yielded mixed findings regarding the amount of self-disclosure elicited by CMC compared with FTF communication [12]. When findings from self-report studies and experimental studies were combined, FTF communication elicited significantly more self-disclosure than CMC (*r*=0.21). However, the association between communication format (FTF vs computer-mediated) and self-disclosure was nonsignificant when analyses were restricted to findings derived solely from true experimental designs (*r*=0.06, *P*>.05).

### MI Language to Predict Changes in Drug Use

Amrhein et al [13] found that the language used by individuals during motivational interviews can help predict which individuals will change a target behavior and which individuals will resist behavior change. Language statements that convey a commitment to behavior change are referred to as “change talk,” and language statements that convey resistance to behavior change are referred to as “sustain talk”. Amrhein’s system for coding commitment language assesses both types of statements [13]. “Change talk” is categorized into six types of statements addressing behavior change, including the desire for behavior change (eg, “I want to cut back on using marijuana”), ability (eg, “I’m capable of living without marijuana”), reasons (eg, “I’m going to lose my kids”), need (eg, “I need to reduce my use”), readiness (eg, “I’m ready to reduce my use”), and commitment (eg, “I swear I will never use marijuana”) to change their behavior (Amrhein PC et al, unpublished data, 2015) [13]. “Sustain talk” is also categorized into six types of statements reflecting a client’s desire, ability, reasons, need, readiness, and commitment to maintaining the target behavior.

Each distinct statement within a motivational interview (also referred to as a “language unit”) is also coded for its “strength” (valence) using scale values ranging from +5 to –5. Positive values reflect the degree to which a statement supports reducing or abstaining from a target behavior (eg, drug use). Negative values reflect the degree to which a statement supports maintaining a target behavior. For example, the statement “I will probably quit” is assigned a valence of +2, whereas the statement “There is no doubt about it; I will quit” is assigned a valence of +5. The latter statement reflects a stronger commitment to changing the target behavior. The use (and strength) of “change talk” during motivational interviews can sometimes predict subsequent behavior change [13-16].

### Marijuana Use in Young Adults

Marijuana use is high among young adults aged 19-30 years. In 2022, 43.6% of a national sample of young adults reported using marijuana during the 12 months preceding the survey, and 28.8% reported using marijuana during the 30 days preceding the survey [17]. The national conversation regarding the legalization of recreational marijuana use may encourage non–marijuana users and occasional marijuana users to consider increasing their own marijuana use, seeking out legal opportunities to use marijuana by visiting states where marijuana use is legal, or seeking out illegal opportunities to use marijuana where recreational marijuana is illegal.

### This Study

This study compared the language content of computer-mediated MTIs and FTF MTIs in young adults who were ambivalent about their level of marijuana use. Non–marijuana users, occasional marijuana users, and frequent marijuana users were recruited to discuss their ambivalence regarding their marijuana use. This study was prompted by the national trend toward the legalization of recreational marijuana use, which is likely to encourage many current nonusers, occasional users, and frequent users to reevaluate their marijuana use. Therefore, non–marijuana users, occasional marijuana users, and frequent marijuana users were recruited to obtain a sample with different levels of marijuana use.

Participants were randomly assigned to receive either a computer-mediated MTI or an FTF MTI. A 2-month follow-up survey assessed their marijuana use during the 2-month period following the interview. We hypothesized that FTF MTIs would elicit more words than computer-mediated MTIs but take less time to complete. We also hypothesized that participants who used language denoting a strong commitment to reduce their marijuana use would report significantly less marijuana use at the 2-month follow-up compared with participants whose MI-type interviews contained weaker commitment language, regardless of interview format (FTF or computer-mediated). Finally, we hypothesized that FTF MTIs and computer-mediated MTIs would elicit the same amount of sustain talk and change talk (eg, desire, ability, reasons, need, commitment, and readiness statements). The latter hypothesis was exploratory because no previous research has compared these two formats for conducting MTIs.

## Methods

### Participants

A total of 150 university students, aged 18-29 (mean 21.3, SD 2.7) years participated in the study. Of these, 52.7% (79/150) were male. Approximately 83.3% (125/150) of participants were Hispanic, 5.3% (8/150) were non-Hispanic White, 5.3% (8/150) were African American, and 6% (9/150) were classified as “other.” These sample demographics reflect the demographics of the university.

A total of 3 types of adults were recruited: non–marijuana users, occasional marijuana users, and frequent marijuana users. Non–marijuana users (n=47) were defined as individuals who reported no history of marijuana use in their lifetime. Occasional marijuana users (n=47) were defined as individuals who used marijuana 1-5 times during the 2 months preceding the study but fewer than 24 times during the year preceding the study. Frequent marijuana users (n=50) were defined as individuals who used marijuana more than 7 times during the 2 months preceding the study and more than 24 times during the year preceding the study. In addition, 6 marijuana users reported no marijuana use during the year preceding the study; therefore, they were classified as lapsed marijuana users. The first 3 categories (ie, non–marijuana users, occasional marijuana users, and frequent marijuana users) were used during the recruitment of participants to obtain a sample with different levels of marijuana use.

To be eligible for the study, young adults had to express ambivalence about their level of marijuana use, as determined by responses to several items on the eligibility survey described below in the Eligibility Assessment section. Young adults were recruited from a large urban university in the Southwestern United States. Recruitment flyers were posted at several locations throughout the university campus.

### Design and Procedure

**Figure 1:**
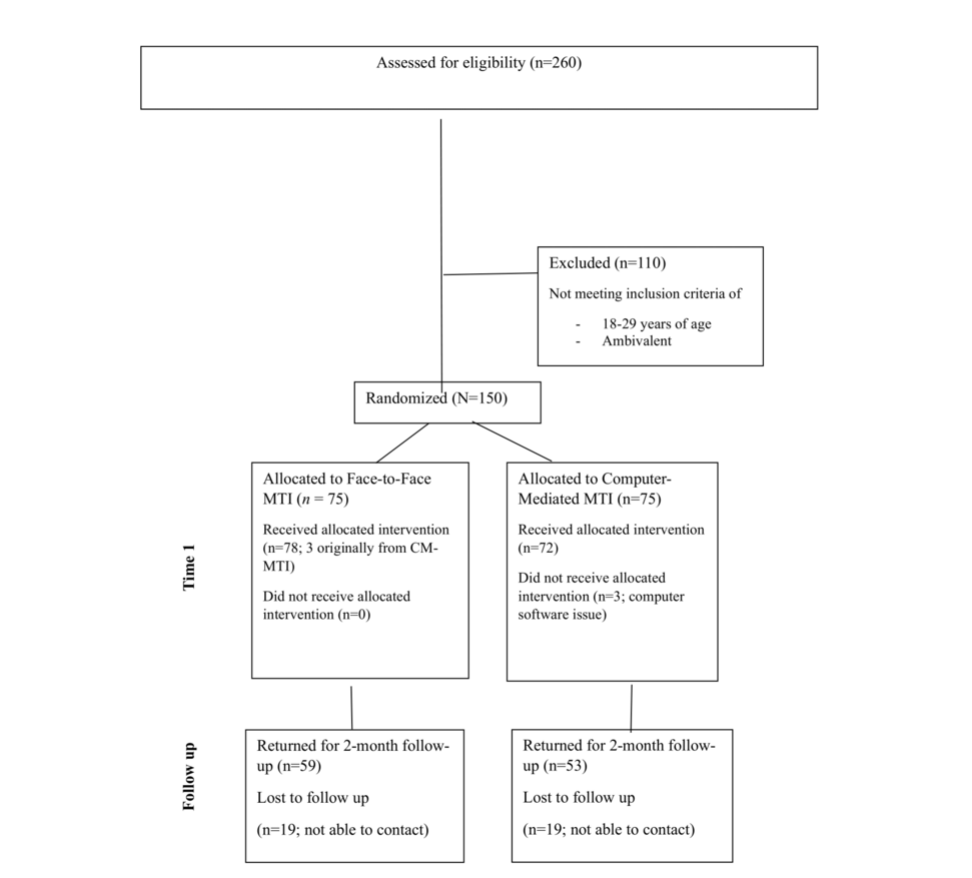
CONSORT (Consolidated Standards of Reporting Trials) diagram showing the flow of participants through Time 1 (baseline assessment) and Time 2 (follow-up assessment).

A between-participants (FTF MTI vs computer-mediated MTI) repeated measures design was used. After assessing eligibility, young adults were randomly assigned using Excel’s (Microsoft Corp) random function to participate in either an FTF MTI (n=75) or a computer-mediated MTI (n=75) at Time 1 (Figure 1). Both types of interviews were guided by the identical interview script. Of these, 3 participants who were originally assigned to the computer-mediated condition completed FTF interviews due to a computer software problem (the interviewer’s prompt did not appear on the participants’ screen and thus, these interviews were completed FTF). Questionnaires administered at Time 1 (baseline assessment: May 2015-October 2015) and Time 2 (follow-up assessment: July 2015-December 2015) assessed the frequency of marijuana use during the 2-month period preceding each assessment.

### Measures and Materials

Participants completed several measures and questionnaires, which are listed below.

#### Eligibility Assessment

A brief eligibility questionnaire assessed each respondent’s age, previous marijuana use, and ambivalence about their marijuana use during the year preceding the study. Sample ambivalence items included: “During the past year I’ve had mixed emotions about my level of marijuana use or nonuse,” and “How much have you thought about changing your marijuana use or nonuse during the past year?” Responses options ranged from 0 (“not at all”) to 5 (“medium amount”) to 10 (“a lot”). Respondents who were 18-29 years of age and reported at least a “medium amount” of ambivalence in response to 2 or more ambivalence questions were eligible to participate in the study.

#### Time 1 Assessment

Eligible participants completed a brief demographic questionnaire as well as a drug use questionnaire assessing the frequency of marijuana use during the two months preceding the study. Response options ranged from “0 times” to “more than 50 times” (sample item: “During the past two months, approximately how many times (if any) have you smoked or consumed marijuana?”). The latter single-item measure served as the key dependent variable. Participants also completed items assessing lifetime use of alcohol, cigarettes, and marijuana, with items adapted from the Monitoring the Future survey [17]. Finally, participants completed tasks assessing their perceived risk of driving a motor vehicle under the combined influence of small amounts of marijuana and alcohol. These latter assessments about driving under the influence were part of a related study conducted by the principal investigators and are not reported here.

#### Time 2 Assessment (2-Month Follow-Up)

The same drug use questionnaire administered at Time 1 was also administered at Time 2, two months following the Time 1 assessment.

#### Brief Motivational-Type Interviews

The interviews conducted in this study were guided by 4 of the 5 major principles of MI [2]. Specifically, the interviews (1) were nonjudgmental, (2) were empathic, (3) respected participants’ autonomy, and (4) helped participants explore their ambivalence toward behavior change (by inviting at least two self-reflections for every question posed to the participant). However, unlike standard motivational interviews, the interviews in this study did not subtly guide participants toward reducing marijuana use. The interviews in this study were not intended as a clinical intervention. Instead, the interviews were conducted to determine if the 2 interview formats (FTF and computer-mediated) (1) elicited similar information and (2) were equally predictive of marijuana use at 2 months post interview. The decision to omit the direction-oriented component of MI was guided by a single consideration: the national trend toward the legalization of recreational marijuana use. For this reason, the interviewer adopted a neutral role, helping participants freely explore their ambivalence about either increasing or decreasing their recreational marijuana use without favoring a specific behavioral outcome. Therefore, these interviews were labeled “MTIs.”

#### FTF Motivational-Type Interviews

FTF MTIs were guided by a 4-page script that incorporated the main principles of MI, including reflective listening (specifically, inviting at least two reflections for each question posed), expression of empathy, and a nonjudgmental conversational style (Miller and Rolnick [2]). The script included an equal number of open-ended questions exploring both the benefits and costs of using marijuana. FTF MTIs were conducted in a research office, and audio was recorded for later transcription.

#### Computer-Mediated MTIs

Computer-mediated interviews were conducted using the identical 4-page script that guided the FTF MTIs. Computer-mediated MTIs were completed via computer, with the interviewer and participant located in adjacent rooms within the same research suite used for the FTF MTIs. However, the interviewer and interviewee never met in person. Upon arrival, a research assistant greeted participants, administered Time 1 assessments, and provided instructions for using the computer’s instant messaging software to communicate during the motivational-type interview. LAN Instant Messenger software (version 1.2.35, Qualia Digital Solutions) was used to conduct computer-mediated interviews and computer-mediated MTIs were saved as text files.

#### Training Motivational Interviewers

Three doctoral assistants received training in MI during a two-day workshop conducted by an experienced member of the Motivational Interviewing Network of Trainers. The doctoral trainees also watched 6 hours of MI training videos [18]. Following this, they participated in role-playing MI sessions and received feedback on their MI skills.

#### Transcribing FTF and Computer-Mediated MTIs

FTF interviews were audio recorded, transcribed into text files, and proofread. Each interview was transcribed by 1 of the 5 undergraduate research assistants. To ensure confidentiality, identifying information that participants inadvertently provided during the interviews, such as the name of a participant’s high school, was deleted. Transcribers also deleted any information from transcripts that could reveal the interview format (FTF or computer-mediated), such as the sounds “um,” “ah,” or “uh,” which participants produced during FTF MTIs.

Computer-mediated MTIs automatically produced written transcripts. To help mask the interview format (FTF or computer-mediated MTIs), the font size, and font type of all transcripts were reformatted to be identical by the same 5 undergraduate students. Typed utterances (eg, uh, um) and symbols (eg, emojis) were also deleted from transcripts to prevent revealing the interview format. Transcribers were unaware of the study’s hypotheses but were aware that 2 types of interviews were conducted (ie, FTF vs computer-mediated MTIs).

#### Coding Commitment Language in MTI Transcripts

For 120 transcripts, each was divided into a series of independent statements identified by a senior team member (KDL, JA, and LDC). Each independent statement represented a unique thought or emotion expressed by the interviewee. Using Amrhein’s coding manual [19], 2 raters independently assigned each verbal statement to 1 of 6 categories that denoted an interviewee’s desire, ability, reason, need, readiness, or commitment to maintain or change their current marijuana use. A seventh category (labeled as “not coded”) was used when a verbal statement could not be assigned to any of the preceding 6 categories. When 2 independent raters could not agree on a final rating of a statement, a third rater was used to reach a consensus and establish the final rating. Statements indicating increasing marijuana use were categorized as sustain talk, while statements indicating decreasing or not using marijuana were categorized as change talk. Sustain talk for nonusers referred to statements indicating increasing marijuana use. Examples of sustain talk for nonusers included: “I want to try marijuana,” or “I will try marijuana on my next trip to Colorado.” Change talk for nonusers referred to statements indicating not using marijuana. Examples of change talk for nonusers included “I will not try marijuana,” “I will not use marijuana,” or “I am worried about trying marijuana.”

Each independent statement within an interview was also coded for its strength (valence). Specifically, each statement was assigned a numeric value between –5 to +5. A negative value denoted a statement that encouraged marijuana use, and a positive value denoted a statement that encouraged the reduction or cessation of marijuana use. Two research assistants independently coded each verbal statement. When the 2 independent raters could not agree on a final rating of a statement, a third rater was used to reach a consensus and establish a final rating. This approach was used to avoid bias and prevented arbitrarily choosing rater 1’s ratings when rater 2 did not agree, or vice versa.

The remaining 30 interviews were segmented using a slightly different procedure to determine how often independent coders identified the same language statements within a transcript. Each pair of coders was initially asked to independently read a transcript and identify (ie, parse) all language statements (units). After segmenting an interview into a series of independent language statements, the 2 laboratory members independently coded each language statement they identified into 1 of 6 categories that denoted an interviewee’s desire, ability, reason, need, readiness, or commitment to maintain or change their current marijuana use. The same coding manual was used to code the remaining 30 interviews. However, we used 2 different strategies for segmenting each MI into a series of independent thoughts or statements (ie, a single reader segmented the interviews into a series of independent language statements for the 120 interviews, while 2 readers segmented the 30 interviews into a series of independent language statements). The degree to which each pair of raters identified the same language statements within these 30 interviews will not be analyzed or reported here because we encountered numerous problems when we tried to compare the language units identified by each pair of coders. For example, the first rater in a pair may have parsed a portion of the interview into the following language unit: “That evening, I decided I would definitely stop using marijuana.” In contrast, the second rater may have omitted the words “that evening” and just parsed the language segment to read: “I decided I would definitely stop using marijuana.” Should these 2 language segments be considered identical? Such complexities led us to abandon the possibility of having a pair of raters parse each interview. The final ratings used for analyses (Mann-Whitney nonparametric tests comparing language use between experimental conditions and negative binomial regression models predicting marijuana use) for these 30 interviews were determined after discussion sessions among both raters. If agreement was not achieved, a third rater decided on the parsed language statements (usually minor words not included in language statements) and ratings.

### Procedure

After completing Time 1 (baseline) assessments of marijuana use, participants were randomly assigned to complete either an FTF MTI or a computer-mediated MTI regarding their current level of marijuana use and associated ambivalence. Participants created a self-generated ID number to ensure the anonymity of their responses while still permitting subsequent pairing of Time 1 and Time 2 assessments. Participants were paid US $20 for completing Time 1 assessments and an additional US $30 for completing Time 2 assessments.

Due to nonnormality violations, Mann-Whitney nonparametric tests were used to compare sustain talk and change talk between the two experimental conditions. Negative binomial regression models were used to predict marijuana use.

### Ethical Considerations

This study was reviewed and approved by the University of Texas at El Paso Institutional Review Board (approval number 219115-2). All participants read and provided informed consent before proceeding with the survey and random assignment to an experimental condition. Participants were assured of confidentiality and created a self-generated ID number to ensure the anonymity of responses while permitting subsequent pairing of Time 1 (baseline) and Time 2 (follow-up) assessments.

## Results

### Commitment Language: Interrater Reliability

Interrater reliability was evaluated based on ratings of 120 out of 150 transcripts as noted earlier. Each transcript was preparsed for utterances and then independently coded by 2 raters. Intraclass correlation coefficient (ICC) for single-item, 2-way random, absolute agreement design were computed for overall category agreement between raters assigning verbal statements to each of the six language categories (ie, Commitment, Desire, Ability, Readiness, Reasons, and Need) or left uncoded, ICC (2,1)=0.78. ICCs were also computed for strength agreement on utterances coded with the same category by the raters. The overall coded categories for strength agreement between raters were ICC (2,1)=0.84, and for each category: Commitment, ICC (2,1)=0.60; Desire, ICC (2,1)=0.81; Ability, ICC (2,1)=0.83; Readiness, ICC (2,1)=0.95; Reasons, ICC (2,1)=0.87; and Need, ICC (2,1)=0.93. Collectively, the ICC values obtained indicate “good” to “excellent” agreement among the raters [20].

### MTI Format, Word Count, and Interview Length

FTF MTIs were completed in significantly less time than computer-mediated MTIs (mean 12.6, SD 5.8 and mean 37.52, SD 9.5 min, respectively; t_143_=–19.25, *P*<.001). However, word count was significantly higher in FTF MTIs compared with computer-mediated MTIs (mean 2011, SD 786.55 and 1015.7, SD 282.54, respectively; t_143_=9.88, *P*<.001, *d*=1.64). Similarly, the mean number of independent language units was significantly higher in FTF MTIs than in computer-mediated MTIs (mean 98.3, SD 46.90 and mean 50, SD 17.57, respectively; t_143_=27.98, *P*<.001, *d*=1.33).

### MTI Format and Commitment Language

FTF MTIs elicited significantly more statements denoting personal reasons for reducing marijuana use (change talk) and more statements denoting a commitment to decrease marijuana use (change talk) compared with computer-mediated MTIs (Table 1). FTF MTIs also elicited significantly more statements expressing both a respondent’s readiness and desire to decrease their marijuana use (change talk) compared to computer-mediated MTIs. However, these two comparisons were not significant after applying Bonferroni corrections for multiple statistical tests (Table 1). In addition, FTF MTIs elicited significantly more statements denoting reasons for marijuana use (sustain talk) and more statements denoting a commitment to use marijuana (Table 1).

Conceivably, FTF MTIs may have encouraged participants to verbalize their thoughts more than computer-mediated MTIs, thereby eliciting significantly more change talk and sustain talk than computer-mediated MTIs. To investigate this possibility, a second set of analyses was conducted, controlling for verbosity. Specifically, we computed the proportion of independent statements (language units) in each transcript that were assigned to overall change talk, sustain talk, and Amrhein’s six commitment language categories (eg, reasons, desires; Table 2). We used a Bonferroni correction for multiple statistical tests (Table 2). After controlling for verbosity, FTF MTIs and computer-mediated MTIs did not differ statistically in the overall amount of change talk (*U*=2435.5, z=–0.72, *P*=.47) and sustain talk (*U*=3110.05, z=1.95, *P*=.05). Similarly, most of the significant language differences in the distinct language categories between FTF MTIs and computer-mediated MTIs disappeared, except for the “not coded” category (Table 2). Language statements assigned to the “not coded” category were slightly higher in the FTF MTIs compared with the computer-mediated MTIs (median 0.14, IQR 0.09-0.21 vs median 0.10, IQR 0.07-0.16, respectively; *U*=1850.5, z=–3.04, *P*=.002).

**Table 1:** Frequency of using each language category within face-to-face and computer-mediated motivational-type interviews.

Dependent variable	FTF^a^ MTI^b^, median (IQR)	CM^c^ MTI, median (IQR)	*U*	z score	*P* value	*d*
Not coded^d^	12 (7.5-19)	4.5 (3-9)	1011.5	–6.38	<.001^d,e^	–1.25
Neutral	3 (2-6)	2 (1-4)	1721.5	–3.59	<.001^d,e^	–0.62
Desire (ST)^f^	1 (0-3)	1 (0-2)	2229	–1.6	.11	–0.27
Ability (ST)	1 (0-3)	1 (0-1)	2065	–2.3	.02^d^	–0.39
Reasons (ST)	21 (16.5-33)	13.5 (10-19.75)	1259.5	–5.39	<.001^d,e^	–1
Need (ST)	0 (0-0)	0 (0-0)	2353.5	–2.05	.04^d^	–0.35
Commitment (ST)	11 (7-18)	7 (4-10)	1429	–4.72	<.001^d,e^	–0.85
Readiness (ST)	0 (0-0)	0 (0-0)	2571.5	–0.36	.72	–0.06
Desire (CT)^g^	2 (0.5-4)	1 (0-2)	1868.5	–3.06	.002^d^	–0.53
Ability (CT)	1 (0-2.5)	0.5 (0-2)	2459	–0.67	.5	–0.11
Reasons (CT)	19 (12-27.50)	8 (5.25-13)	945	–6.63	<.001^d,e^	–1.32
Need (CT)	0 (0-1)	0 (0-0)	2350.5	–1.42	.16	–0.24
Commitment (CT)	7 (4-9)	3.5 (2-6)	1583.5	–4.11	<.001^d,e^	–0.73
Readiness (CT)	0 (0-0)	0 (0-0)	2281.5	–2.79	.005^d^	–0.48

^a^FTF: face-to-face.

^b^Five motivational-type interview transcripts were lost during the transcription process.

^c^CM: computer-mediated.

^d^Statistically significant at *P*<.05.

^e^Statistically significant results after a Bonferroni correction (0.05/14 tests=0.004).

^f^ST: sustain talk.

^g^CT: change talk.

**Table 2:** Controlling for verbosity: proportion of language statements in each language category for face-to-face and computer-mediated motivational-type interviews.

Dependent variable	FTF^a^ MTI^b,^ median (IQR)	CM^c^ MTI, median (IQR)	*U*	z score	*P* value	*d*
Not coded^d^	0.14^d^ (0.09-0.21)	0.10^d^ (0.07-0.16)	1850.5^d^	–3.04^d^	.002^d,e^	–0.52^d^
Neutral	0.04 (0.02-0.06)	0.04 (0.02-0.07)	2794	0.7	.49	0.12
Desire (ST)^f^	0.02 (0-0.03)	0.02 (0-0.04)	2689.5	0.29	.77	0.05
Ability (ST)	0.01 (0-0.03)	0.01 (0-0.02)	2436	–0.75	.45	–0.12
Reasons (ST)	0.27 (0.21-0.33)	0.28 (0.22-0.38)	2915.5	1.18	.24	0.2
Need (ST)	0 (0-0)	0 (0-0)	2367	–1.94	.05	–0.33
Commitment (ST)	0.13 (0.09-0.17)	0.14 (0.09-0.20)	2908	1.15	.25	0.19
Readiness (ST)	0 (0-0)	0 (0-0)	2591	–0.21	.84	–0.03
Desire (CT)^g^	0.02 (0-0.04)	0.02 (0-0.05)	2388.5	–0.93	.35	–0.15
Ability (CT)	0.01 (0-0.03)	0.01 (0-0.04)	2862.5	1.03	.3	0.17
Reasons (CT)	0.21^d^ (0.15-0.27)	0.19^d^ (0.12-0.24)	2066.5^d^	–2.19^d^	.03^d^	–0.37^d^
Need (CT)	0 (0-0.01)	0 (0-0)	2432.5	–0.98	.33	–0.16
Commitment (CT)	0.07 (0.05-0.10)	0.07 (0.03-0.14)	2811.5	0.77	.44	0.13
Readiness (CT)	0^d^ (0-0)	0^d^ (0-0)	2283^d^	–2.78^d^	.005^d^	–0.47^d^

^a^FTF: face-to-face.

^b^Five motivational-type interview transcripts were lost during the transcription process.

^c^CM: computer-mediated.

^d^Statistically significant at *P*<.05.

^e^Statistically significant results after a Bonferroni correction (0.05/14 tests=0.004).

^f^ST: sustain talk.

^g^CT: change talk.

### MTI Format and Strength of Commitment Language

We computed six strength variables for desire, ability, reasons, needs, commitment, and readiness statements. For instance, the “strength of commitment” variable was calculated by summing the positive and negative values assigned to each commitment statement within an interview. The latter sum was then divided by the number of commitment statements within an interview, yielding a “strength of commitment” variable. Computer-mediated MTIs elicited significantly stronger statements denoting “reasons” for encouraging marijuana use than did FTF MTIs (mean valence ratings –0.48, SD 0.85 and –0.08, SD 0.95, respectively; t_143_=2.63, *P*=.01, *d*=0.44). However, the latter difference was not significant after using a Bonferroni correction for six multiple comparisons (*P*<.008).

### Motivational-Type Interview Format (Computer-Mediated vs FTF), Commitment Language, and Behavior Change

A total of 75% (n=112) of Time 1 participants returned for 2-month follow-up assessments. The frequency of marijuana use at Time 1 was not significantly different between participants in the FTF MTI and computer-mediated MTI conditions (mean 10.49, SD 15.60 and mean 8.73, SD 16.61; t_147_=0.67, *P*=.50, *d*=0.11). Frequency of marijuana use at Time 1 was not significantly different between participants lost at follow-up and participants who returned for a 2-month follow-up (mean 6.18, SD 12.09 and mean 11.23, SD 16.91; t_147_=–1.99, *P*=.09, *d*=0.34). After controlling for marijuana use at Time 1, the strength of commitment language at Time 1 and marijuana use at Time 2 was not statistically significant, semipartial correlation *r*=0.03, *P*=.57. Frequency of marijuana use at the 2-month follow-up was not significantly different between participants in the FTF MTI and computer-mediated MTI conditions (mean 12.44, SD 18.01 and mean 9.98, SD 17.22; t_110_=0.74, *P*=.46, *d*=0.14). We used a negative binomial regression with a log link function to test if the relationship between Time 2 marijuana use and the strength of Time 1 commitment language depended upon the type of interview that was conducted (ie, FTF MTI vs computer-mediated MTI). Both gender and Time 1 marijuana use were entered as covariates (Table 3). Experimental condition (ie, FTF MTI or computer-mediated MTI) did not predict Time 2 marijuana use (B=0.45, *P*=.13). Similarly, the strength of commitment language at Time 1 did not predict Time 2 marijuana use (B*=*–0.25, *P*=.08). However, the interaction between experimental condition and the strength of Time 1 commitment language did predict Time 2 marijuana use (B=0.45, *P*=.007). This finding suggests that the relationship between the strength of commitment language at Time 1 and frequency of marijuana use at Time 2 varies depending on the MTI format: FTF MTI versus computer-mediated MTI.

**Table 3:** Negative binomial models predicting the frequency of marijuana use at Time 2. The main model includes 106 participants with valid observations for all variables in the model. Dependent variable question: “During the past two months, how many times (if any) have you smoked or consumed marijuana?”

Predictors		Β^a^	SE	OR^b^ (95% CI)
Intercept		0.04	0.28	1.04 (0.60-1.82)
Sex (male=0, female=1)		0.40	0.28	1.49 (0.85-2.61)
Baseline marijuana use^c^		0.09	0.01	1.09 (1.07-1.11)
Condition (FTF^d^=0, CM^e^=1)		0.45	0.30	1.57 (0.87-2.81)
Strength of commitment language		–0.25	0.14	0.78 (0.60-1.03)
Condition*strength of commitment language^c^		0.45	0.17	1.57 (1.13-2.18)
Scale		1	—^f^	—
Negative binomial^g^		1.53	0.30	—
**FTF negative binomial model predicting frequency of marijuana use at Time 2**				
	Intercept	0.03	0.30	1.03 (0.57-1.84)
	Gender (male=0, female=1)	0.27	0.32	1.31 (0.70-2.46)
	Baseline marijuana use^c^	0.09	0.01	1.10 (1.07-1.12)
	Strength of commitment language	–0.21	0.12	0.89 (0.64-1.03)
	Scale	1	—	—
	Negative binomial	1.05	0.29	—
**CM negative binomial model predicting frequency of marijuana use at Time 2**				
	Intercept	0.45	0.4	1.57 (0.72-3.41)
	Gender (male=0, female=1)	0.64	0.51	1.89 (0.70-5.15)
	Baseline marijuana use^c^	0.07	0.02	1.07 (1.04-1.11)
	Strength of commitment language	0.13	0.14	1.14 (0.87-1.48)
	Scale	1	—	—
	Negative binomial	2.13	0.60	—

^a^Β: negative binomial regression coefficient.

^b^OR: odds ratio.

^c^Statistically significant at *P*<.05.

^d^FTF: face-to-face motivational interviews.

^e^CM: computer-mediated.

^f^Not applicable.

^g^The negative binomial model is more appropriate than the Poisson model, as indicated by the dispersion coefficient test.

We probed the above interaction between experimental condition and the strength of Time 1 commitment language further, again using negative binomial regression with a log link function to model the relationship between Time 1 commitment language and Time 2 marijuana use. We entered gender and Time 1 marijuana use as covariates in the analysis**.** Based on data solely from participants in the FTF MTI condition, Time 2 marijuana use appeared to decrease as the strength of Time 1 commitment language increased, although this finding was not significant (B=–0.21, *P*=.08). However, participants in the computer-mediated MTI condition displayed a different pattern of findings: Time 2 marijuana use was not significantly related to the strength of Time 1 commitment language (B=0.13, *P*=.35). The potential implication of this finding is discussed in the discussion section below.

A second set of analyses was conducted excluding nonusers and rerunning the negative binomial model predicting marijuana use at the 2-month follow-up. After excluding nonusers, we entered gender, experimental condition, the strength of commitment language, and the interaction between commitment language and condition. We found a similar pattern to the overall sample (Table 3) and the analyses that exclude nonusers of marijuana. The interaction between experimental conditions and the strength of Time 1 commitment language did predict Time 2 marijuana use (B=0.38, *P*=.02). We probed the interaction using negative binomial regression with a log link function to model the relationship between Time 1 commitment language and Time 2 marijuana use for each of the experimental conditions. For participants in the FTF MTI condition, Time 2 marijuana use appeared to decrease as the strength of Time 1 commitment language increased. However, this finding was not significant (B=–0.15, *P*=.20). For participants in the computer-mediated MTI condition, Time 2 marijuana use appeared to increase as the strength of Time 1 commitment language increased. However, this finding was not significant (B=0.14, *P*=.29).

## Discussion

### Principal Findings

This is the first study to compare the language used by individuals during computer-mediated MTIs versus FTF MTIs. Notably, the current findings reveal that computer-mediated MTIs elicit both similar amounts of overall change talk and sustain talk, key elements of traditional FTF motivational interviews. If future studies confirm the current findings, then computer-mediated MTIs may eventually provide an evidence-based alternative to FTF interviews when clients and other interviewees are unable to meet physically with a counselor, clinician, or other type of interviewer. Indeed, computer-mediated MTIs may have several benefits, including the ability to reach traditionally inaccessible or underserved populations who would benefit from MTI interventions targeting a range of health-threatening behaviors, such as alcohol and tobacco use [4,21].

Although the current findings suggest that computer-mediated MTIs are feasible, this study revealed several important differences between the language used by interviewees during computer-mediated and FTF MTIs. First, FTF MTIs elicited significantly more material from interviewees. Specifically, FTF MTIs elicited significantly more words and independent statements from respondents during the interviews. In addition, computer-mediated MTIs required significantly more time to complete an interview compared with FTF interviews. Notably, even after controlling for the amount of material elicited during FTF MTIs, participants in FTF MTIs provided a significantly greater proportion of statements denoting reasons for decreasing their own marijuana use. Participants in FTF MTIs also produced a significantly greater proportion of statements denoting their readiness to decrease their own marijuana use. The latter finding suggests that computer-mediated MTIs may benefit from the inclusion of additional verbal prompts designed to elicit more material from interviewees. However, these latter differences were not statistically significant after using a conservative Bonferroni correction. Future studies would benefit from investigating this issue further. Language statements assigned to the “not coded” category were slightly higher in the FTF MTIs compared with the computer-mediated MTIs. The MTIs may have been influenced by our prompt to discuss personal marijuana use, especially given that many states were legalizing the recreational use of marijuana at the time. Some participants wanted to discuss general issues regarding the legalization of marijuana. Therefore, these statements were coded as “not coded” if a participant’s response focused on their opinions about “legalization” rather than their personal marijuana use. We considered these general statements about the legalization of marijuana as a way to build rapport and comfort in discussing their marijuana use. We should also note that the current findings regarding the number of “not coded” statements in our sample are not entirely inconsistent with other findings [16,22]. For example, in a sample of 92 motivational interviews, Apodaca and colleagues coded 21% of the language statements as “neutral or follow” and 16% of the statements as “change talk” [22].

Several studies of MI suggest that behavior change can be predicted, in part, by the type of language that participants use when discussing their future behavior. Statements denoting a strong commitment to reducing unwanted behavior (ie, change talk) have been associated with subsequent behavior change in MI participants [13-16]. In this study, we found that the direction of the relationship may depend on the type of interview in which individuals participate: computer-mediated versus FTF. In addition, the relationship between change talk and Time 2 marijuana use disappeared when we controlled for Time 1 marijuana use.

A total of 3 explanations could account for our failure to detect a significant relationship between change talk and Time 2 marijuana use among participants in either experimental condition (ie, computer-mediated MTI and FTF MTI). First, we conducted subgroup analyses, examining the relationship between change talk and Time 2 marijuana use for participants in the computer-mediated MTIs and participants in the FTF MTIs. A loss of statistical power was associated with each subgroup analysis. Smaller sample sizes within each experimental condition make it more difficult to detect significant relationships among variables when those relationships exist in the population. Second, and more intriguingly, computer-mediated MTIs may impact behavior differently than FTF MTIs. Third, both conditions may elicit relatively low commitment strength because of the lack of a directionality component. Alternatively, these findings may reflect an artifact of the way this variable (strength of commitment) was computed. Specifically, we summed the positive and negative values assigned to each commitment statement within an interview. The latter sum was then divided by the number of commitment statements within an interview, yielding a “strength of commitment” variable. It is possible that adding negative and positive values may mask important relationships when aggregating this type of variable, rather than looking at commitment language throughout an interview (ie, beginning, middle, and end of the interview). Future research would benefit from exploring this issue. The current findings suggest that computer-mediated and FTF MTIs both elicit key aspects of change talk (eg, statements denoting reasons for change, desire to change, and commitment to change). However, it is possible that computer-mediated MTIs do not result in behavior change. Future research will need to investigate this issue with larger participant samples and longer assessment periods.

Several features of this study increased the internal validity of our findings. First, participants were randomly assigned to the interview format (FTF and computer-mediated MTIs), thereby increasing internal validity. Second, identical MTI scripts were used to guide FTF MTIs and computer-mediated MTIs, again increasing the internal validity of the findings. Third, several strategies were used to maintain blind coding of each interview, helping to mask a participant’s experimental condition (FTF MTI vs computer-mediated MTI) and thus minimize bias when identifying commitment language within each interview transcript. Fourth, every transcript was coded by 2 independent raters.

Despite the above strengths of the study design, the current findings have limited applicability to the immediate practice of MI that uses a text-based format. The study was not designed as a clinical intervention. Therefore, the text-based interviews and FTF interviews conducted in this study did not selectively evoke or reinforce change talk in participants, a key component of MI [5,23]. Instead, participants were invited to explore their ambivalence about their own recreational marijuana use without interviewers seeking to influence the behavior of participants. These conversations took place with interviewers who were trained in the spirit of MI and instructed to be (1) nonjudgmental, (2) empathic, and (3) respectful of each participant’s autonomy. From this standpoint, the marijuana-related interviews conducted in this study were characterized by the “relational” component of MI and several “technical” components (eg, reflections, affirmations, and open-ended questions). The relational components of MI emphasize the “skill of accurate empathy” and the spirit of MI, while one of the technical components emphasizes the elicitation of “change talk” [5]. A 2017 meta-analysis of 19 MI studies revealed a significant association between an interviewer’s increasing use of MI’s relational components and a client’s increasing use of both change talk and sustain talk (*r*=0.25 and 0.18, respectively) [24]. The latter finding suggests that empathic interviewers who are guided by the spirit of MI can elicit change talk from individuals even in the absence of selectively evoking or reinforcing such talk (ie, the directional component). Findings from this study add to the latter research by comparing the amount of change talk and sustain talk elicited in text-based MI-type interviews and FTF MI-type interviews. Both change talk and sustain talk were elicited from participants in the text-based interviews conducted in this study. However, change talk was not significantly associated with an individual’s self-reported marijuana use at Time 2 follow-up. The latter finding is consistent with the results of two meta-analytic reviews that revealed no association between clinical outcome and empathy or MI spirit [23,24].

Although the text-based interviews and FTF interviews in this study did not selectively reinforce change talk, both interview formats incorporated several technical components of MI, including the use of simple reflections, complex reflections, and affirmations. The latter components may have also contributed to the ability of the text-based interviews to elicit change talk.

As noted earlier, this study was prompted by the national trend toward the legalization of recreational marijuana use. This trend will likely encourage many “nonusers” and “occasional users” to reevaluate their marijuana use. We sought to determine if a brief MI-type interview could elicit language (eg, change talk) that predicted behavior change in adults who were ambivalent about their limited marijuana use. Such information could be useful for developing interventions that encourage responsible marijuana use in states that legalize recreational use. For this reason, we purposely omitted selective reinforcement of sustain talk (among nonusers) and change talk (among occasional users). However, from an ethical standpoint, we could not justify subtly manipulating the behavior of participants who were not seeking treatment for drug use nor were engaged in health-threatening behavior that was legal in an increasing number of states.

Several aspects of this study were exploratory. First, MI is often used with individuals who are engaged in health-threatening behaviors. In contrast, this study used MI-type interviews with adults who were ambivalent about their limited use of marijuana. Second, the study sought to determine if text-based MI-type interviews and FTF MI-type interviews elicited the same language (eg, change talk). Since our MI-type interviews did not selectively reinforce change talk, this study might best be characterized as a preliminary investigation of “communication modality in discussions of health behavior” rather than a study of MI per se. The current findings have implications for health-related practitioners.

This research design may partially limit the internal validity and external validity of the current findings. First, we could not completely mask the format of the interview (FTF vs computer-mediated) that generated each interview and associated transcript. For example, the cadence of speech differs from the cadence of writing, and thus even when all relevant words were deleted from a transcript, coders could conceivably identify the interview format (computer-mediated vs FTF), at least in some instances. Second, we did not assess the fidelity of the interviews; that is, we did not evaluate the extent to which the interviews themselves were consistent with the principles of MI. Although the script provided to each interviewer was purposely designed to incorporate MI principles, we did not evaluate the extent to which the interviewers, themselves, followed the script as instructed. Third, the marijuana-related interviews conducted in this study did not specifically aim to reduce a participant’s marijuana use. Instead, participants were encouraged to explore their ambivalence about their marijuana use, an issue that we discussed earlier in this paper. This modification could have influenced the type of commitment language used by participants during the interview. This modification may also have influenced the impact of commitment language on Time 2 marijuana use. Finally, this study used a single item to assess the frequency of marijuana use at Time 1 and Time 2 assessments. A single-item assessment may be subject to several recall biases that could be overcome by also obtaining biomarkers of marijuana use. Future research would benefit from investigating these issues.

This study suggests several additional avenues for future research. First, future research would benefit from replacing our use of MTIs with standard motivational interviews, where the interviewer intentionally guides the interview toward a targeted behavior (eg, decreasing drug use and increasing exercise). Second, future studies would benefit from using experienced motivational interviewers to help determine the external validity of the current findings. The importance of identifying alternative computer-mediated strategies for conducting traditional FTF motivational interviews was highlighted during the COVID-19 pandemic. For example, an April 2020 national probability survey revealed that an estimated 75% of the US population had “completely or mostly isolated themselves from people outside of their household” [25]. Such isolation restricted opportunities for participating in FTF motivational interviews, highlighting the need to study the benefits and limitations of conducting computer-mediated motivational interviews.

### Conclusion

This paper is one of the first studies to compare the language used by individuals during computer-mediated MTIs versus FTF MTIs. Computer-mediated and FTF MTIs elicit both change talk and sustain talk. This indicates that motivational interviews could be adapted for delivery via text-based computer platforms. Yet, further research is needed to enhance the predictive validity of the type of language obtained via computer-delivered MI.

## Appendix

Multimedia Appendix 1 CONSORT-eHEALTH checklist (V 1.6.1).

## Abbreviations

CMC: computer-mediated communication
FTF: face-to-face
ICC: intraclass correlation coefficient
MI: motivational interviewing
MTI: motivational-type interview

## Data Availability

The datasets generated or analyzed during this study are available from the corresponding author on reasonable request.
