# Supplementary material for: Impact of Computer-Mediated Versus Face-to-Face Motivational-Type Interviews on Participants’ Language and Subsequent Cannabis Use: Randomized Controlled Trial
Source: J Med Internet Res. 2025 Apr 25;27:e59085. doi: 10.2196/59085 (PMC12064966; doi:10.2196/59085)
Supplement: Multimedia Appendix 1 [file jmir_v27i1e59085_app1.pdf]

CONSORT-EHEALTH (V 1.6.1) - Submission/Publication Form

The CONSORT-EHEALTH checklist is intended for authors of randomized trials evaluating web-based and Internet-based applications/interventions, including mobile interventions, electronic games (incl multiplayer games), social media, certain telehealth applications, and other interactive and/or networked electronic applications. Some of the items (e.g. all subitems under item 5 - description of the intervention) may also be applicable for other study designs.

The goal of the CONSORT EHEALTH checklist and guideline is to be
a) a guide for reporting for authors of RCTs,
b) to form a basis for appraisal of an ehealth trial (in terms of validity)

CONSORT-EHEALTH items/subitems are MANDATORY reporting items for studies published in the Journal of Medical Internet Research and other journals / scientific societies endorsing the checklist.

Items numbered 1., 2., 3., 4a., 4b etc are original CONSORT or CONSORT-NPT (non-pharmacologic treatment) items.
Items with Roman numerals (i., ii, iii, iv etc.) are CONSORT-EHEALTH extensions/clarifications.

As the CONSORT-EHEALTH checklist is still considered in a formative stage, we would ask that you also RATE ON A SCALE OF 1-5 how important/useful you feel each item is FOR THE PURPOSE OF THE CHECKLIST and reporting guideline (optional).

Mandatory reporting items are marked with a red *.
In the textboxes, either copy & paste the relevant sections from your manuscript into this form - please include any quotes from your manuscript in QUOTATION MARKS,
or answer directly by providing additional information not in the manuscript, or elaborating on why the item was not relevant for this study.

YOUR ANSWERS WILL BE PUBLISHED AS A SUPPLEMENTARY FILE TO YOUR PUBLICATION IN JMIR AND ARE CONSIDERED PART OF YOUR PUBLICATION (IF ACCEPTED).
Please fill in these questions diligently. Information will not be copyedited, so please use proper spelling and grammar, use correct capitalization, and avoid abbreviations.

DO NOT FORGET TO SAVE AS PDF _AND_ CLICK THE SUBMIT BUTTON SO YOUR ANSWERS ARE IN OUR DATABASE !!!

Citation Suggestion (if you append the pdf as Appendix we suggest to cite this paper in the caption):
Eysenbach G, CONSORT-EHEALTH Group

CONSORT-EHEALTH: Improving and Standardizing Evaluation Reports of Web-based and Mobile Health Interventions
J Med Internet Res 2011;13(4):e126
URL: http://www.jmir.org/2011/4/e126/
doi: 10.2196/jmir.1923
PMID: 22209829

kdllanes2011@gmail.com Switch account

Not shared

* Indicates required question

Your name *

First Last

Karla Llanes

Primary Affiliation (short), City, Country *

University of Toronto, Toronto, Canada

University of California, San Francisco, USA

Your e-mail address *

abc@gmail.com

karla.llanes@ucsf.edu

Title of your manuscript *

Provide the (draft) title of your manuscript.

Impact of Computer-Mediated Versus Face-to-Face Motivational-Type Interviews on Participant's Language and Subsequent Cannabis Use: Randomized Controlled Trial

Name of your App/Software/Intervention *

If there is a short and a long/alternate name, write the short name first and add the long name in brackets.

Computer-Mediated Motivational Type Intervie

Evaluated Version (if any)

e.g. "V1", "Release 2017-03-01", "Version 2.0.27913"

Not Applicable

Language(s) *

What language is the intervention/app in? If multiple languages are available, separate by comma (e.g. "English, French")

English

URL of your Intervention Website or App

e.g. a direct link to the mobile app on app in appstore (itunes, Google Play), or URL of the website. If the intervention is a DVD or hardware, you can also link to an Amazon page.

Your answer

URL of an image/screenshot (optional)

Your answer

Accessibility *

Can an enduser access the intervention presently?

- ○ access is free and open
- ○ access only for special usergroups, not open
- ○ access is open to everyone, but requires payment/subscription/in-app purchases
- ○ app/intervention no longer accessible
- ◉ Other: The intervention was only available to participants enrolled in study.

Primary Medical Indication/Disease/Condition *

e.g. "Stress", "Diabetes", or define the target group in brackets after the condition, e.g. "Autism (Parents of children with)", "Alzheimers (Informal Caregivers of)"

Cannabis use

Primary Outcomes measured in trial *

comma-separated list of primary outcomes reported in the trial

Experimental differences in change talk and su

Secondary/other outcomes

Are there any other outcomes the intervention is expected to affect?

We did not examine secondary outcomes.

Recommended "Dose" *

What do the instructions for users say on how often the app should be used?

- ○ Approximately Daily
- ○ Approximately Weekly
- ○ Approximately Monthly
- ○ Approximately Yearly
- ○ "as needed"
- ◉ Other: Participants received either one brief face-to-face motivational type in

Approx. Percentage of Users (starters) still using the app as recommended after 3 months *

- ◉ unknown / not evaluated
- ○ 0-10%
- ○ 11-20%
- ○ 21-30%
- ○ 31-40%
- ○ 41-50%
- ○ 51-60%
- ○ 61-70%
- ○ 71%-80%
- ○ 81-90%
- ○ 91-100%
- ○ Other:

Overall, was the app/intervention effective? *

- ○ yes: all primary outcomes were significantly better in intervention group vs control
- ○ partly: SOME primary outcomes were significantly better in intervention group vs control
- ◉ no statistically significant difference between control and intervention
- ○ potentially harmful: control was significantly better than intervention in one or more outcomes
- ○ inconclusive: more research is needed
- ○ Other: ______

Article Preparation Status/Stage *

At which stage in your article preparation are you currently (at the time you fill in this form)

- ○ not submitted yet - in early draft status
- ○ not submitted yet - in late draft status, just before submission
- ○ submitted to a journal but not reviewed yet
- ○ submitted to a journal and after receiving initial reviewer comments
- ◉ submitted to a journal and accepted, but not published yet
- ○ published
- ○ Other: ______

Journal *

If you already know where you will submit this paper (or if it is already submitted), please provide the journal name (if it is not JMIR, provide the journal name under "other")

- ○ not submitted yet / unclear where I will submit this
- ◉ Journal of Medical Internet Research (JMIR)
- ○ JMIR mHealth and UHealth
- ○ JMIR Serious Games
- ○ JMIR Mental Health
- ○ JMIR Public Health
- ○ JMIR Formative Research
- ○ Other JMIR sister journal
- ○ Other:

Is this a full powered effectiveness trial or a pilot/feasibility trial? *

- ○ Pilot/feasibility
- ◉ Fully powered

Manuscript tracking number *

If this is a JMIR submission, please provide the manuscript tracking number under "other" (The ms tracking number can be found in the submission acknowledgement email, or when you login as author in JMIR. If the paper is already published in JMIR, then the ms tracking number is the four-digit number at the end of the DOI, to be found at the bottom of each published article in JMIR)

- ○ no ms number (yet) / not (yet) submitted to / published in JMIR
- ◉ Other: JMIR ms#59085

TITLE AND ABSTRACT

1a) TITLE: Identification as a randomized trial in the title

1a) Does your paper address CONSORT item 1a? *

I.e does the title contain the phrase "Randomized Controlled Trial"? (if not, explain the reason under "other")

- (x) yes
- ( ) Other:

1a-i) Identify the mode of delivery in the title

Identify the mode of delivery. Preferably use “web-based” and/or “mobile” and/or “electronic game” in the title. Avoid ambiguous terms like “online”, “virtual”, “interactive”. Use “Internet-based” only if Intervention includes non-web-based Internet components (e.g. email), use “computer-based” or “electronic” only if offline products are used. Use “virtual” only in the context of “virtual reality” (3-D worlds). Use “online” only in the context of “online support groups”. Complement or substitute product names with broader terms for the class of products (such as “mobile” or “smart phone” instead of “iphone”), especially if the application runs on different platforms.

	1	2	3	4	5	
subitem not at all important	( )	( )	( )	( )	(x)	essential

Clear selection

Does your paper address subitem 1a-i? *

Copy and paste relevant sections from manuscript title (include quotes in quotation marks "like this" to indicate direct quotes from your manuscript), or elaborate on this item by providing additional information not in the ms, or briefly explain why the item is not applicable/relevant for your study

Impact of Computer-Mediated Versus Face-to-Face Motivational-Type Interviews on Participant's Language and Subsequent Cannabis Use: Randomized Controlled Trial

1a-ii) Non-web-based components or important co-interventions in title

Mention non-web-based components or important co-interventions in title, if any (e.g., "with telephone support").

	1	2	3	4	5	
subitem not at all important	○	○	○	○	◉	essential

Clear selection

Does your paper address subitem 1a-ii?

Copy and paste relevant sections from manuscript title (include quotes in quotation marks "like this" to indicate direct quotes from your manuscript), or elaborate on this item by providing additional information not in the ms, or briefly explain why the item is not applicable/relevant for your study

Computer-mediated interviews were conducted using the identical 4-page script that guided the FTF MTIs. Computer-mediated MTIs were completed via computer, with the interviewer and participant located in adjacent rooms within the same research suite used for the FTF MTIs. However, the interviewer and interviewee never met in person. Upon arrival, a research assistant greeted participants, administered Time 1 assessments, and provided instructions for using the computer's instant messaging software to communicate during the motivational-type interview. LAN Instant Messenger software (version 1.2.35, Qualia Digital Solutions) was used to conduct computer-mediated interviews and computer-mediated MTIs were saved as text files.

1a-iii) Primary condition or target group in the title

Mention primary condition or target group in the title, if any (e.g., “for children with Type I Diabetes”) Example: A Web-based and Mobile Intervention with Telephone Support for Children with Type I Diabetes: Randomized Controlled Trial

	1	2	3	4	5	
subitem not at all important	(●)	( )	( )	( )	( )	essential

Clear selection

Does your paper address subitem 1a-iii? *

Copy and paste relevant sections from manuscript title (include quotes in quotation marks "like this" to indicate direct quotes from your manuscript), or elaborate on this item by providing additional information not in the ms, or briefly explain why the item is not applicable/relevant for your study

We describe our sample consisted of young adults in the methods section.

1b) ABSTRACT: Structured summary of trial design, methods, results, and conclusions

NPT extension: Description of experimental treatment, comparator, care providers, centers, and blinding status.

1b-i) Key features/functionalities/components of the intervention and comparator in the METHODS section of the ABSTRACT

Mention key features/functionalities/components of the intervention and comparator in the abstract. If possible, also mention theories and principles used for designing the site. Keep in mind the needs of systematic reviewers and indexers by including important synonyms. (Note: Only report in the abstract what the main paper is reporting. If this information is missing from the main body of text, consider adding it)

	1	2	3	4	5	
subitem not at all important	○	○	○	○	◉	essential

Clear selection

Does your paper address subitem 1b-i? *

Copy and paste relevant sections from the manuscript abstract (include quotes in quotation marks "like this" to indicate direct quotes from your manuscript), or elaborate on this item by providing additional information not in the ms, or briefly explain why the item is not applicable/relevant for your study

Background: Motivational interviewing (MI) is frequently used to facilitate behavior change. The use of change talk during motivational interviews can predict subsequent behavior change. However, no studies have compared the information obtained from traditional face-to-face motivational interviews and computer-mediated motivational interviews or resulted in the same amount of behavior change. Objective: This study aimed to investigate if face-to-face motivational-type interviews (MTIs) and computer-mediated MTIs elicit the same amount of “change talk” and behavior change when young adults discuss their ambivalence about using marijuana. Methods: A total of 150 users, including frequent marijuana users, occasional marijuana users, and non–marijuana users, participated in the study. All participants reported being at least moderately ambivalent about their current level of marijuana use. Participants were randomly assigned to complete a brief MTI using either the standard face-to-face format or a computer-mediated format. Amrhein’s manual for assessing the presence of “change talk” and “sustain talk” was used to code the language produced by respondents in each interview format. A reduction in marijuana use was asse

1b-ii) Level of human involvement in the METHODS section of the ABSTRACT

Clarify the level of human involvement in the abstract, e.g., use phrases like “fully automated” vs. “therapist/nurse/care provider/physician-assisted” (mention number and expertise of providers involved, if any). (Note: Only report in the abstract what the main paper is reporting. If this information is missing from the main body of text, consider adding it)

	1	2	3	4	5	
subitem not at all important	○	○	○	○	○	essential

Does your paper address subitem 1b-ii?

Copy and paste relevant sections from the manuscript abstract (include quotes in quotation marks "like this" to indicate direct quotes from your manuscript), or elaborate on this item by providing additional information not in the ms, or briefly explain why the item is not applicable/relevant for your study

Methods: A total of 150 users, including frequent marijuana users, occasional marijuana users, and non–marijuana users, participated in the study. All participants reported being at least moderately ambivalent about their current level of marijuana use. Participants were randomly assigned to complete a brief MTI using either the standard face-to-face format or a computer-mediated format.

1b-iii) Open vs. closed, web-based (self-assessment) vs. face-to-face assessments in the METHODS section of the ABSTRACT

Mention how participants were recruited (online vs. offline), e.g., from an open access website or from a clinic or a closed online user group (closed usergroup trial), and clarify if this was a purely web-based trial, or there were face-to-face components (as part of the intervention or for assessment). Clearly say if outcomes were self-assessed through questionnaires (as common in web-based trials). Note: In traditional offline trials, an open trial (open-label trial) is a type of clinical trial in which both the researchers and participants know which treatment is being administered. To avoid confusion, use “blinded” or “unblinded” to indicated the level of blinding instead of “open”, as “open” in web-based trials usually refers to “open access” (i.e. participants can self-enrol). (Note: Only report in the abstract what the main paper is reporting. If this information is missing from the main body of text, consider adding it)

	1	2	3	4	5	
subitem not at all important	○	○	○	○	◉	essential

Clear selection

Does your paper address subitem 1b-iii?

Copy and paste relevant sections from the manuscript abstract (include quotes in quotation marks "like this" to indicate direct quotes from your manuscript), or elaborate on this item by providing additional information not in the ms, or briefly explain why the item is not applicable/relevant for your study

Due to word limit, we provide this information in the method section.

1b-iv) RESULTS section in abstract must contain use data

Report number of participants enrolled/assessed in each group, the use/uptake of the intervention (e.g., attrition/adherence metrics, use over time, number of logins etc.), in addition to primary/secondary outcomes. (Note: Only report in the abstract what the main paper is reporting. If this information is missing from the main body of text, consider adding it)

	1	2	3	4	5	
subitem not at all important	○	○	○	○	◉	essential

Clear selection

Does your paper address subitem 1b-iv?

Copy and paste relevant sections from the manuscript abstract (include quotes in quotation marks "like this" to indicate direct quotes from your manuscript), or elaborate on this item by providing additional information not in the ms, or briefly explain why the item is not applicable/relevant for your study

A total of 150 users, including frequent marijuana users, occasional marijuana users, and non–marijuana users, participated in the study. All participants reported being at least moderately ambivalent about their current level of marijuana use. Participants were randomly assigned to complete a brief MTI using either the standard face-to-face format or a computer-mediated format.

1b-v) CONCLUSIONS/DISCUSSION in abstract for negative trials

Conclusions/Discussions in abstract for negative trials: Discuss the primary outcome - if the trial is negative (primary outcome not changed), and the intervention was not used, discuss whether negative results are attributable to lack of uptake and discuss reasons. (Note: Only report in the abstract what the main paper is reporting. If this information is missing from the main body of text, consider adding it)

	1	2	3	4	5	
subitem not at all important	○	○	○	○	◉	essential

Clear selection

Does your paper address subitem 1b-v?

Copy and paste relevant sections from the manuscript abstract (include quotes in quotation marks "like this" to indicate direct quotes from your manuscript), or elaborate on this item by providing additional information not in the ms, or briefly explain why the item is not applicable/relevant for your study

The word count was significantly higher in face-to-face MTIs compared with computer-mediated MTIs (P<.001). After controlling for verbosity, face-to-face MTIs, and computer-mediated MTIs did not differ statistically in the overall amount of change talk (P=.47) and sustain talk (P=.05). Face-to-face MTIs elicited significantly more reasons for reducing future marijuana use (ie, change talk; P=.02) and readiness toward not using marijuana (ie, change talk; P=.009), even after controlling for verbosity. However, these differences were not statistically significant after using a conservative Bonferroni correction (P<.004). After controlling for marijuana use at Time 1, the relationship between the strength of commitment language at Time 1 and marijuana use at Time 2 was not statistically significant (semipartial correlation r=0.03, P=.57). The association between Time 1 change talk and Time 2 marijuana use depended on the type of motivational interview that participants experienced: face-to-face MTI versus computer-mediated MTI (B=0.45, P=.01). A negative binomial regression with a log link function was used to probe this relationship after controlling for 2 covariates: gender and Time 1 (baseline assessment) marijuana use. Among participants in the face-to-face MTI condition, Time 2 (follow-up) marijuana use decreased as the strength of Time 1 change talk increased, although this finding was not significant (B=−0.21, P=.08). However, among participants in the computer-mediated MTI condition, Time 2 marijuana use was not significantly related to the strength of Time 1 change talk (B=0.13, P=.16). Conclusions: Computer-mediated MTIs and face-to-face MTIs elicit both change talk and sustain talk, which suggests that motivational interviews could potentially be adapted for delivery via text-based computer platforms. However, further research is needed to enhance the predictive validity of the type of language obtained via computer-delivered MI.

INTRODUCTION

2a) In INTRODUCTION: Scientific background and explanation of rationale

2a-i) Problem and the type of system/solution

Describe the problem and the type of system/solution that is object of the study: intended as stand-alone intervention vs. incorporated in broader health care program? Intended for a particular patient population? Goals of the intervention, e.g., being more cost-effective to other interventions, replace or complement other solutions? (Note: Details about the intervention are provided in “Methods” under 5)

	1	2	3	4	5	
subitem not at all important	○	○	○	○	◉	essential

Clear selection

Does your paper address subitem 2a-i? *

Copy and paste relevant sections from the manuscript (include quotes in quotation marks "like this" to indicate direct quotes from your manuscript), or elaborate on this item by providing additional information not in the ms, or briefly explain why the item is not applicable/relevant for your study

Your answer

2a-ii) Scientific background, rationale: What is known about the (type of) system

Scientific background, rationale: What is known about the (type of) system that is the object of the study (be sure to discuss the use of similar systems for other conditions/diagnoses, if appropiate), motivation for the study, i.e. what are the reasons for and what is the context for this specific study, from which stakeholder viewpoint is the study performed, potential impact of findings [2]. Briefly justify the choice of the comparator.

	1	2	3	4	5	
subitem not at all important	○	○	○	○	◉	essential

Clear selection

Does your paper address subitem 2a-ii? *

Copy and paste relevant sections from the manuscript (include quotes in quotation marks "like this" to indicate direct quotes from your manuscript), or elaborate on this item by providing additional information not in the ms, or briefly explain why the item is not applicable/relevant for your study

However, no studies have compared the information obtained from (1) traditional face-to-face (FTF) motivational interviews and (2) technology-assisted MIs. It is not known if FTF MIs and technology-assisted MIs elicit the same number of words and thoughts during the interview process, nor is it known if traditional FTF MIs and technology-assisted MIs elicit the same amount of verbal commitment to changing a target behavior (eg, reducing drug use). Finally, it is not known if FTF MIs and technology-assisted MIs elicit the same amount of change talk, sustain talk, and behavior change. The current research begins to address these gaps in knowledge by comparing the language elicited from participants during FTF motivational-type interviews (MTIs) and text-based (computer-mediated) MTIs.

2b) In INTRODUCTION: Specific objectives or hypotheses

Does your paper address CONSORT subitem 2b? *

Copy and paste relevant sections from the manuscript (include quotes in quotation marks "like this" to indicate direct quotes from your manuscript), or elaborate on this item by providing additional information not in the ms, or briefly explain why the item is not applicable/relevant for your study

This study compared the language content of computer-mediated MTIs and FTF MTIs in young adults who were ambivalent about their level of marijuana use. Non–marijuana users, occasional marijuana users, and frequent marijuana users were recruited to discuss their ambivalence regarding their marijuana use. This study was prompted by the national trend toward the legalization of recreational marijuana use, which is likely to encourage many current nonusers, occasional users, and frequent users to reevaluate their marijuana use. Therefore, non–marijuana users, occasional marijuana users, and frequent marijuana users were recruited to obtain a sample with different levels of marijuana use. Participants were randomly assigned to receive either a computer-mediated MTI or an FTF MTI. A 2-month follow-up survey assessed their marijuana use during the 2-month period following the interview. We hypothesized that FTF MTIs would elicit more words than computer-mediated MTIs but take less time to complete. We also hypothesized that participants who used language denoting a strong commitment to reduce their marijuana use would report significantly less marijuana use at the 2-month follow-up compared with participants whose MI-type interviews contained weaker commitment language, regardless of interview format (FTF or computer-mediated). Finally, we hypothesized that FTF MTIs and computer-mediated MTIs would elicit the same amount of sustain talk and change talk (eg, desire, ability, reasons, need, commitment, and readiness statements). The latter hypothesis was exploratory because no previous research has compared these two formats for conducting MTIs.

METHODS

3a) Description of trial design (such as parallel, factorial) including allocation ratio

Does your paper address CONSORT subitem 3a? *

Copy and paste relevant sections from the manuscript (include quotes in quotation marks "like this" to indicate direct quotes from your manuscript), or elaborate on this item by providing additional information not in the ms, or briefly explain why the item is not applicable/relevant for your study

A between-participants (FTF MTI vs computer-mediated MTI) repeated measures design was used. After assessing eligibility, young adults were randomly assigned using Excel's (Microsoft Corp) random function to participate in either an FTF MTI (n=75) or a computer-mediated MTI (n=75) at Time 1 (Figure 1). Both types of interviews were guided by the identical interview script. Of these, 3 participants who were originally assigned to the computer-mediated condition completed FTF interviews due to a computer software problem (the interviewer's prompt did not appear on the participants' screen and thus, these interviews were completed FTF).

3b) Important changes to methods after trial commencement (such as eligibility criteria), with reasons

Does your paper address CONSORT subitem 3b? *

Copy and paste relevant sections from the manuscript (include quotes in quotation marks "like this" to indicate direct quotes from your manuscript), or elaborate on this item by providing additional information not in the ms, or briefly explain why the item is not applicable/relevant for your study

We did not change methods after trial commenced.

3b-i) Bug fixes, Downtimes, Content Changes

Bug fixes, Downtimes, Content Changes: ehealth systems are often dynamic systems. A description of changes to methods therefore also includes important changes made on the intervention or comparator during the trial (e.g., major bug fixes or changes in the functionality or content) (5-iii) and other “unexpected events” that may have influenced study design such as staff changes, system failures/downtimes, etc. [2].

	1	2	3	4	5	
subitem not at all important	○	○	○	○	◉	essential

Clear selection

Does your paper address subitem 3b-i?

Copy and paste relevant sections from the manuscript (include quotes in quotation marks "like this" to indicate direct quotes from your manuscript), or elaborate on this item by providing additional information not in the ms, or briefly explain why the item is not applicable/relevant for your study

A between-participants (FTF MTI vs computer-mediated MTI) repeated measures design was used. After assessing eligibility, young adults were randomly assigned using Excel’s (Microsoft Corp) random function to participate in either an FTF MTI (n=75) or a computer-mediated MTI (n=75) at Time 1 (Figure 1). Both types of interviews were guided by the identical interview script. "Of these, 3 participants who were originally assigned to the computer-mediated condition completed FTF interviews due to a computer software problem (the interviewer’s prompt did not appear on the participants’ screen and thus, these interviews were completed FTF)".

4a) Eligibility criteria for participants

Does your paper address CONSORT subitem 4a? *

Copy and paste relevant sections from the manuscript (include quotes in quotation marks "like this" to indicate direct quotes from your manuscript), or elaborate on this item by providing additional information not in the ms, or briefly explain why the item is not applicable/relevant for your study

Respondents who were 18-29 years of age and reported at least a “medium amount” of ambivalence in response to 2 or more ambivalence questions were eligible to participate in the study.

4a-i) Computer / Internet literacy

Computer / Internet literacy is often an implicit “de facto” eligibility criterion - this should be explicitly clarified.

	1	2	3	4	5	
subitem not at all important	○	○	●	○	○	essential

Clear selection

Does your paper address subitem 4a-i?

Copy and paste relevant sections from the manuscript (include quotes in quotation marks "like this" to indicate direct quotes from your manuscript), or elaborate on this item by providing additional information not in the ms, or briefly explain why the item is not applicable/relevant for your study

We did not assess internet literacy because we recruited from a large urban university who are likely skilled in computers and smartphones. "Young adults were recruited from a large urban university in the Southwestern United States."

4a-ii) Open vs. closed, web-based vs. face-to-face assessments:

Open vs. closed, web-based vs. face-to-face assessments: Mention how participants were recruited (online vs. offline), e.g., from an open access website or from a clinic, and clarify if this was a purely web-based trial, or there were face-to-face components (as part of the intervention or for assessment), i.e., to what degree got the study team to know the participant. In online-only trials, clarify if participants were quasi-anonymous and whether having multiple identities was possible or whether technical or logistical measures (e.g., cookies, email confirmation, phone calls) were used to detect/prevent these.

	1	2	3	4	5	
subitem not at all important	○	○	○	○	◉	essential

Clear selection

Does your paper address subitem 4a-ii? *

Copy and paste relevant sections from the manuscript (include quotes in quotation marks "like this" to indicate direct quotes from your manuscript), or elaborate on this item by providing additional information not in the ms, or briefly explain why the item is not applicable/relevant for your study

Computer-mediated MTIs were completed via computer, with the interviewer and participant located in adjacent rooms within the same research suite used for the FTF MTIs. However, the interviewer and interviewee never met in person. Upon arrival, a research assistant greeted participants, administered Time 1 assessments, and provided instructions for using the computer’s instant messaging software to communicate during the motivational-type interview. LAN Instant Messenger software (version 1.2.35, Qualia Digital Solutions) was used to conduct computer-mediated interviews and computer-mediated MTIs were saved as text files.

4a-iii) Information giving during recruitment

Information given during recruitment. Specify how participants were briefed for recruitment and in the informed consent procedures (e.g., publish the informed consent documentation as appendix, see also item X26), as this information may have an effect on user self-selection, user expectation and may also bias results.

	1	2	3	4	5	
subitem not at all important	○	○	○	○	●	essential

Clear selection

Does your paper address subitem 4a-iii?

Copy and paste relevant sections from the manuscript (include quotes in quotation marks "like this" to indicate direct quotes from your manuscript), or elaborate on this item by providing additional information not in the ms, or briefly explain why the item is not applicable/relevant for your study

This study was reviewed and approved by the University of Texas at El Paso Institutional Review Board (approval number 219115-2). All participants read and provided informed consent before proceeding with the survey and random assignment to an experimental condition. Participants were assured of confidentiality and created a self-generated ID number to ensure the anonymity of responses while permitting subsequent pairing of Time 1 (baseline) and Time 2 (follow-up) assessments.

4b) Settings and locations where the data were collected

Does your paper address CONSORT subitem 4b? *

Copy and paste relevant sections from the manuscript (include quotes in quotation marks "like this" to indicate direct quotes from your manuscript), or elaborate on this item by providing additional information not in the ms, or briefly explain why the item is not applicable/relevant for your study

Young adults were recruited from a large urban university in the Southwestern United States. Recruitment flyers were posted at several locations throughout the university campus.

4b-i) Report if outcomes were (self-)assessed through online questionnaires

Clearly report if outcomes were (self-)assessed through online questionnaires (as common in web-based trials) or otherwise.

	1	2	3	4	5	
subitem not at all important	○	○	○	○	◉	essential

Clear selection

Does your paper address subitem 4b-i? *

Copy and paste relevant sections from the manuscript (include quotes in quotation marks "like this" to indicate direct quotes from your manuscript), or elaborate on this item by providing additional information not in the ms, or briefly explain why the item is not applicable/relevant for your study

All surveys were administered in-person by a trained research assistant. "Upon arrival, a research assistant greeted participants, administered Time 1 assessments, and provided instructions for using the computer's instant messaging software to communicate during the motivational-type interview. LAN Instant Messenger software (version 1.2.35, Qualia Digital Solutions) was used to conduct computer-mediated interviews and computer-mediated MTIs were saved as text files."

4b-ii) Report how institutional affiliations are displayed

Report how institutional affiliations are displayed to potential participants [on ehealth media], as affiliations with prestigious hospitals or universities may affect volunteer rates, use, and reactions with regards to an intervention.(Not a required item – describe only if this may bias results)

	1	2	3	4	5	
subitem not at all important	○	○	○	○	◉	essential

Clear selection

Does your paper address subitem 4b-ii?

Copy and paste relevant sections from the manuscript (include quotes in quotation marks "like this" to indicate direct quotes from your manuscript), or elaborate on this item by providing additional information not in the ms, or briefly explain why the item is not applicable/relevant for your study

Young adults were recruited from a large urban university in the Southwestern United States. Recruitment flyers were posted at several locations throughout the university campus.

5) The interventions for each group with sufficient details to allow replication, including how and when they were actually administered

5-i) Mention names, credential, affiliations of the developers, sponsors, and owners

Mention names, credential, affiliations of the developers, sponsors, and owners [6] (if authors/evaluators are owners or developer of the software, this needs to be declared in a "Conflict of interest" section or mentioned elsewhere in the manuscript).

	1	2	3	4	5	
subitem not at all important	○	○	○	○	◉	essential

Clear selection

Does your paper address subitem 5-i?

Copy and paste relevant sections from the manuscript (include quotes in quotation marks "like this" to indicate direct quotes from your manuscript), or elaborate on this item by providing additional information not in the ms, or briefly explain why the item is not applicable/relevant for your study

The interviews conducted in this study were guided by 4 of the 5 major principles of MI [2]. Specifically, the interviews (1) were nonjudgmental, (2) were empathic, (3) respected participants'autonomy, and (4) helped participants explore their ambivalence toward behavior change (by inviting at least two self-reflections for every question posed to the participant). However, unlike standard motivational interviews, the interviews in this study did not subtly guide participants toward reducing marijuana use. The interviews in this study were not intended as a clinical intervention. Instead, the interviews were conducted to determine if the 2 interview formats (FTF and computer-mediated) (1) elicited similar information and (2) were equally predictive of marijuana use at 2 months post interview. The decision to omit the direction-oriented component of MI was guided by a single consideration: the national trend toward the legalization of recreational marijuana use. For this reason, the interviewer adopted a neutral role, helping participants freely explore their ambivalence about either increasing or decreasing their recreational marijuana use without favoring a specific behavioral outcome. Therefore, these interviews were labeled "MTIs." FTF Motivational-Type Interviews FTF MTIs were guided by a 4-page script that incorporated the main principles of MI, including reflective listening (specifically, inviting at least two reflections for each question posed), expression of empathy, and a nonjudgmental conversational style (Miller and Rolnick [2]). The script included an equal number of open-ended questions exploring both the benefits and costs of using marijuana. FTF MTIs were conducted in a research office, and audio was recorded for later transcription. Computer-Mediated MTIs Computer-mediated interviews were conducted using the identical 4-page script that guided the FTF MTIs. Computer-mediated MTIs were completed via computer, with the interviewer and participant located in adjacent rooms within the same research suite used for the FTF MTIs. However, the interviewer and interviewee never met in person. Upon arrival, a research assistant greeted participants, administered Time 1 assessments, and provided instructions for using the computer's instant messaging software to communicate during the motivational-type interview. LAN Instant Messenger software (version 1.2.35, Qualia Digital Solutions) was used to conduct computer-mediated interviews and computer-mediated MTIs were saved as text files.

5-ii) Describe the history/development process

Describe the history/development process of the application and previous formative evaluations (e.g., focus groups, usability testing), as these will have an impact on adoption/use rates and help with interpreting results.

	1	2	3	4	5	
subitem not at all important	○	○	○	○	◉	essential

Clear selection

Does your paper address subitem 5-ii?

Copy and paste relevant sections from the manuscript (include quotes in quotation marks "like this" to indicate direct quotes from your manuscript), or elaborate on this item by providing additional information not in the ms, or briefly explain why the item is not applicable/relevant for your study

Three doctoral assistants received training in MI during a two-day workshop conducted by an experienced member of the Motivational Interviewing Network of Trainers. The doctoral trainees also watched 6 hours of MI training videos [18]. Following this, they participated in role-playing MI sessions and received feedback on their MI skills. https://www.jmir.org/2025/1/e59085/ XSL•FO Llanes et al Transcribing FTF and Computer-Mediated MTIs FTF interviews were audio recorded, transcribed into text files, and proofread. Each interview was transcribed by 1 of the 5 undergraduate research assistants. To ensure confidentiality, identifying information that participants inadvertently provided during the interviews, such as the name of a participant's high school, was deleted. Transcribers also deleted any information from transcripts that could reveal the interview format (FTF or computer-mediated), such as the sounds "um," "ah," or "uh," which participants produced during FTF MTIs. Computer-mediated MTIs automatically produced written transcripts. To help mask the interview format (FTF or computer-mediated MTIs), the font size, and font type of all transcripts were reformatted to be identical by the same 5 undergraduate students. Typed utterances (eg, uh, um) and symbols (eg, emojis) were also deleted from transcripts to prevent revealing the interview format. Transcribers were unaware of the study's hypotheses but were aware that 2 types of interviews were conducted (ie, FTF vs computer-mediated MTIs). Coding Commitment Language in MTI Transcripts For 120 transcripts, each was divided into a series of independent statements identified by a senior team member (KDL, JA, and LDC). Each independent statement represented a unique thought or emotion expressed by the interviewee. Using Amrhein's coding manual [19], 2 raters independently assigned each verbal statement to 1 of 6 categories that denoted an interviewee's desire, ability, reason, need, readiness, or commitment to maintain or change their current marijuana use. A seventh category (labeled as "not coded") was used when a verbal statement could not be assigned to any of the preceding 6 categories.

5-iii) Revisions and updating

Revisions and updating. Clearly mention the date and/or version number of the application/intervention (and comparator, if applicable) evaluated, or describe whether the intervention underwent major changes during the evaluation process, or whether the development and/or content was “frozen” during the trial. Describe dynamic components such as news feeds or changing content which may have an impact on the replicability of the intervention (for unexpected events see item 3b).

	1	2	3	4	5	
subitem not at all important	○	○	○	○	◉	essential

Clear selection

Does your paper address subitem 5-iii?

Copy and paste relevant sections from the manuscript (include quotes in quotation marks "like this" to indicate direct quotes from your manuscript), or elaborate on this item by providing additional information not in the ms, or briefly explain why the item is not applicable/relevant for your study

We did not make changes to the interview guide after participants were randomly assigned.

5-iv) Quality assurance methods

Provide information on quality assurance methods to ensure accuracy and quality of information provided [1], if applicable.

	1	2	3	4	5	
subitem not at all important	○	○	○	○	◉	essential

Clear selection

Does your paper address subitem 5-iv?

Copy and paste relevant sections from the manuscript (include quotes in quotation marks "like this" to indicate direct quotes from your manuscript), or elaborate on this item by providing additional information not in the ms, or briefly explain why the item is not applicable/relevant for your study

Three doctoral assistants received training in MI during a two-day workshop conducted by an experienced member of the Motivational Interviewing Network of Trainers. The doctoral trainees also watched 6 hours of MI training videos [18]. Following this, they participated in role-playing MI sessions and received feedback on their MI skills. https://www.jmir.org/2025/1/e59085/ XSL•FO Llanes et al Transcribing FTF and Computer-Mediated MTIs FTF interviews were audio recorded, transcribed into text files, and proofread. Each interview was transcribed by 1 of the 5 undergraduate research assistants. To ensure confidentiality, identifying information that participants inadvertently provided during the interviews, such as the name of a participant's high school, was deleted. Transcribers also deleted any information from transcripts that could reveal the interview format (FTF or computer-mediated), such as the sounds "um," "ah," or "uh," which participants produced during FTF MTIs. Computer-mediated MTIs automatically produced written transcripts. To help mask the interview format (FTF or computer-mediated MTIs), the font size, and font type of all transcripts were reformatted to be identical by the same 5 undergraduate students. Typed utterances (eg, uh, um) and symbols (eg, emojis) were also deleted from transcripts to prevent revealing the interview format. Transcribers were unaware of the study's hypotheses but were aware that 2 types of interviews were conducted (ie, FTF vs computer-mediated MTIs). Coding Commitment Language in MTI Transcripts For 120 transcripts, each was divided into a series of independent statements identified by a senior team member (KDL, JA, and LDC). Each independent statement represented a unique thought or emotion expressed by the interviewee. Using Amrhein's coding manual [19], 2 raters independently assigned each verbal statement to 1 of 6 categories that denoted an interviewee's desire, ability, reason, need, readiness, or commitment to maintain or change their current marijuana use. A seventh category (labeled as "not coded") was used when a verbal statement could not be assigned to any of the preceding 6 categories. W

5-v) Ensure replicability by publishing the source code, and/or providing screenshots/screen-capture video, and/or providing flowcharts of the algorithms used

Ensure replicability by publishing the source code, and/or providing screenshots/screen-capture video, and/or providing flowcharts of the algorithms used. Replicability (i.e., other researchers should in principle be able to replicate the study) is a hallmark of scientific reporting.

	1	2	3	4	5	
subitem not at all important	○	○	○	○	◉	essential

Clear selection

Does your paper address subitem 5-v?

Copy and paste relevant sections from the manuscript (include quotes in quotation marks "like this" to indicate direct quotes from your manuscript), or elaborate on this item by providing additional information not in the ms, or briefly explain why the item is not applicable/relevant for your study

We provide a reference with survey items and interview guide questions.

5-vi) Digital preservation

Digital preservation: Provide the URL of the application, but as the intervention is likely to change or disappear over the course of the years; also make sure the intervention is archived (Internet Archive, webcitation.org, and/or publishing the source code or screenshots/videos alongside the article). As pages behind login screens cannot be archived, consider creating demo pages which are accessible without login.

	1	2	3	4	5	
subitem not at all important	◉	○	○	○	○	essential

Clear selection

Does your paper address subitem 5-vi?

Copy and paste relevant sections from the manuscript (include quotes in quotation marks "like this" to indicate direct quotes from your manuscript), or elaborate on this item by providing additional information not in the ms, or briefly explain why the item is not applicable/relevant for your study

The computer-mediated motivational type interview was only available to participants enrolled in the study.

5-vii) Access

Access: Describe how participants accessed the application, in what setting/context, if they had to pay (or were paid) or not, whether they had to be a member of specific group. If known, describe how participants obtained “access to the platform and Internet” [1]. To ensure access for editors/reviewers/readers, consider to provide a “backdoor” login account or demo mode for reviewers/readers to explore the application (also important for archiving purposes, see vi).

	1	2	3	4	5	
subitem not at all important	○	○	○	○	◉	essential

Clear selection

Does your paper address subitem 5-vii? *

Copy and paste relevant sections from the manuscript (include quotes in quotation marks "like this" to indicate direct quotes from your manuscript), or elaborate on this item by providing additional information not in the ms, or briefly explain why the item is not applicable/relevant for your study

Computer-mediated interviews were conducted using the identical 4-page script that guided the FTF MTIs. Computer-mediated MTIs were completed via computer, with the interviewer and participant located in adjacent rooms within the same research suite used for the FTF MTIs. However, the interviewer and interviewee never met in person. Upon arrival, a research assistant greeted participants, administered Time 1 assessments, and provided instructions for using the computer's instant messaging software to communicate during the motivational-type interview. LAN Instant Messenger software (version 1.2.35, Qualia Digital Solutions) was used to conduct computer-mediated interviews and computer-mediated MTIs were saved as text files.

5-viii) Mode of delivery, features/functionalities/components of the intervention and comparator, and the theoretical framework

Describe mode of delivery, features/functionalities/components of the intervention and comparator, and the theoretical framework [6] used to design them (instructional strategy [1], behaviour change techniques, persuasive features, etc., see e.g., [7, 8] for terminology). This includes an in-depth description of the content (including where it is coming from and who developed it) [1]," whether [and how] it is tailored to individual circumstances and allows users to track their progress and receive feedback" [6]. This also includes a description of communication delivery channels and – if computer-mediated communication is a component – whether communication was synchronous or asynchronous [6]. It also includes information on presentation strategies [1], including page design principles, average amount of text on pages, presence of hyperlinks to other resources, etc. [1].

	1	2	3	4	5	
subitem not at all important	○	○	○	○	◉	essential

Clear selection

Does your paper address subitem 5-viii? *

Copy and paste relevant sections from the manuscript (include quotes in quotation marks "like this" to indicate direct quotes from your manuscript), or elaborate on this item by providing additional information not in the ms, or briefly explain why the item is not applicable/relevant for your study

Computer-mediated interviews were conducted using the identical 4-page script that guided the FTF MTIs. Computer-mediated MTIs were completed via computer, with the interviewer and participant located in adjacent rooms within the same research suite used for the FTF MTIs. However, the interviewer and interviewee never met in person. Upon arrival, a research assistant greeted participants, administered Time 1 assessments, and provided instructions for using the computer's instant messaging software to communicate during the motivational-type interview. LAN Instant Messenger software (version 1.2.35, Qualia Digital Solutions) was used to conduct computer-mediated interviews and computer-mediated MTIs were saved as text files.

5-ix) Describe use parameters

Describe use parameters (e.g., intended "doses" and optimal timing for use). Clarify what instructions or recommendations were given to the user, e.g., regarding timing, frequency, heaviness of use, if any, or was the intervention used ad libitum.

	1	2	3	4	5	
subitem not at all important	○	○	○	○	●	essential

Clear selection

Does your paper address subitem 5-ix?

Copy and paste relevant sections from the manuscript (include quotes in quotation marks "like this" to indicate direct quotes from your manuscript), or elaborate on this item by providing additional information not in the ms, or briefly explain why the item is not applicable/relevant for your study

A between-participants (FTF MTI vs computer-mediated MTI) repeated measures design was used. After assessing eligibility, young adults were randomly assigned using Excel's (Microsoft Corp) random function to participate in either an FTF MTI (n=75) or a computer-mediated MTI (n=75) at Time 1 (Figure 1). Both types of interviews were guided by the identical interview script. Of these, 3 participants who were originally assigned to the computer-mediated condition completed FTF interviews due to a computer software problem (the interviewer's prompt did not appear on the participants' screen and thus, these interviews were completed FTF). Questionnaires administered at Time 1 (baseline assessment: May 2015-October 2015) and Time 2 (follow-up assessment: July 2015-December 2015) assessed the frequency of marijuana use during the 2-month period preceding each assessment.

5-x) Clarify the level of human involvement

Clarify the level of human involvement (care providers or health professionals, also technical assistance) in the e-intervention or as co-intervention (detail number and expertise of professionals involved, if any, as well as "type of assistance offered, the timing and frequency of the support, how it is initiated, and the medium by which the assistance is delivered". It may be necessary to distinguish between the level of human involvement required for the trial, and the level of human involvement required for a routine application outside of a RCT setting (discuss under item 21 – generalizability).

	1	2	3	4	5	
subitem not at all important	○	○	○	○	◉	essential

Clear selection

Does your paper address subitem 5-x?

Copy and paste relevant sections from the manuscript (include quotes in quotation marks "like this" to indicate direct quotes from your manuscript), or elaborate on this item by providing additional information not in the ms, or briefly explain why the item is not applicable/relevant for your study

Computer-mediated interviews were conducted using the identical 4-page script that guided the FTF MTIs. Computer-mediated MTIs were completed via computer, with the interviewer and participant located in adjacent rooms within the same research suite used for the FTF MTIs. However, the interviewer and interviewee never met in person. Upon arrival, a research assistant greeted participants, administered Time 1 assessments, and provided instructions for using the computer's instant messaging software to communicate during the motivational-type interview. LAN Instant Messenger software (version 1.2.35, Qualia Digital Solutions) was used to conduct computer-mediated interviews and computer-mediated MTIs were saved as text files.

Three doctoral assistants received training in MI during a two-day workshop conducted by an experienced member of the Motivational Interviewing Network of Trainers. The doctoral trainees also watched 6 hours of MI training videos [18]. Following this, they participated in role-playing MI sessions and received feedback on their MI skills.

5-xi) Report any prompts/reminders used

Report any prompts/reminders used: Clarify if there were prompts (letters, emails, phone calls, SMS) to use the application, what triggered them, frequency etc. It may be necessary to distinguish between the level of prompts/reminders required for the trial, and the level of prompts/reminders for a routine application outside of a RCT setting (discuss under item 21 – generalizability).

	1	2	3	4	5	
subitem not at all important	(●)	( )	( )	( )	( )	essential

Clear selection

Does your paper address subitem 5-xi? *

Copy and paste relevant sections from the manuscript (include quotes in quotation marks "like this" to indicate direct quotes from your manuscript), or elaborate on this item by providing additional information not in the ms, or briefly explain why the item is not applicable/relevant for your study

Participants only received one brief motivational type (computer mediated or face-to-face) interview session (15-45 minutes).

5-xii) Describe any co-interventions (incl. training/support)

Describe any co-interventions (incl. training/support): Clearly state any interventions that are provided in addition to the targeted eHealth intervention, as ehealth intervention may not be designed as stand-alone intervention. This includes training sessions and support [1]. It may be necessary to distinguish between the level of training required for the trial, and the level of training for a routine application outside of a RCT setting (discuss under item 21 – generalizability.

	1	2	3	4	5	
subitem not at all important	○	○	○	○	●	essential

Clear selection

Does your paper address subitem 5-xii? *

Copy and paste relevant sections from the manuscript (include quotes in quotation marks "like this" to indicate direct quotes from your manuscript), or elaborate on this item by providing additional information not in the ms, or briefly explain why the item is not applicable/relevant for your study

Three doctoral assistants received training in MI during a two-day workshop conducted by an experienced member of the Motivational Interviewing Network of Trainers. The doctoral trainees also watched 6 hours of MI training videos [18]. Following this, they participated in role-playing MI sessions and received feedback on their MI skills.

6a) Completely defined pre-specified primary and secondary outcome measures, including how and when they were assessed

Does your paper address CONSORT subitem 6a? *

Copy and paste relevant sections from the manuscript (include quotes in quotation marks "like this" to indicate direct quotes from your manuscript), or elaborate on this item by providing additional information not in the ms, or briefly explain why the item is not applicable/relevant for your study

The same drug use questionnaire administered at Time 1 was also administered at Time 2, two months following the Time 1 assessment.

6a-i) Online questionnaires: describe if they were validated for online use and apply CHERRIES items to describe how the questionnaires were designed/deployed

If outcomes were obtained through online questionnaires, describe if they were validated for online use and apply CHERRIES items to describe how the questionnaires were designed/deployed [9].

	1	2	3	4	5	
subitem not at all important	○	○	○	○	◉	essential

Clear selection

Does your paper address subitem 6a-i?

Copy and paste relevant sections from manuscript text

All surveys were administered by a trained research assistant and not online.

6a-ii) Describe whether and how "use" (including intensity of use/dosage) was defined/measured/monitored

Describe whether and how "use" (including intensity of use/dosage) was defined/measured/monitored (logins, logfile analysis, etc.). Use/adoption metrics are important process outcomes that should be reported in any ehealth trial.

	1	2	3	4	5	
subitem not at all important	○	○	○	○	◉	essential

Clear selection

Does your paper address subitem 6a-ii?

Copy and paste relevant sections from manuscript text

A between-participants (FTF MTI vs computer-mediated MTI) repeated measures design was used. After assessing eligibility, young adults were randomly assigned using Excel's (Microsoft Corp) random function to participate in either an FTF MTI (n=75) or a computer-mediated MTI (n=75) at Time 1 (Figure 1). Both types of interviews were guided by the identical interview script. Of these, 3 participants who were originally assigned to the computer-mediated condition completed FTF interviews due to a computer software problem (the interviewer's prompt did not appear on the participants' screen and thus, these interviews were completed FTF). Questionnaires administered at Time 1 (baseline assessment: May 2015-October 2015) and Time 2 (follow-up assessment: July 2015-December 2015) assessed the frequency of marijuana use during the 2-month period preceding each assessment.

6a-iii) Describe whether, how, and when qualitative feedback from participants was obtained

Describe whether, how, and when qualitative feedback from participants was obtained (e.g., through emails, feedback forms, interviews, focus groups).

	1	2	3	4	5	
subitem not at all important	◉	○	○	○	○	essential

Clear selection

Does your paper address subitem 6a-iii?

Copy and paste relevant sections from manuscript text

No

6b) Any changes to trial outcomes after the trial commenced, with reasons

Does your paper address CONSORT subitem 6b? *

Copy and paste relevant sections from the manuscript (include quotes in quotation marks "like this" to indicate direct quotes from your manuscript), or elaborate on this item by providing additional information not in the ms, or briefly explain why the item is not applicable/relevant for your study

no

7a) How sample size was determined

NPT: When applicable, details of whether and how the clustering by care provides or centers was addressed

7a-i) Describe whether and how expected attrition was taken into account when calculating the sample size

Describe whether and how expected attrition was taken into account when calculating the sample size.

	1	2	3	4	5	
subitem not at all important	○	○	○	○	○	essential

Does your paper address subitem 7a-i?

Copy and paste relevant sections from manuscript title (include quotes in quotation marks "like this" to indicate direct quotes from your manuscript), or elaborate on this item by providing additional information not in the ms, or briefly explain why the item is not applicable/relevant for your study

Your answer

7b) When applicable, explanation of any interim analyses and stopping guidelines

Does your paper address CONSORT subitem 7b? *

Copy and paste relevant sections from the manuscript (include quotes in quotation marks "like this" to indicate direct quotes from your manuscript), or elaborate on this item by providing additional information not in the ms, or briefly explain why the item is not applicable/relevant for your study

Your answer

8a) Method used to generate the random allocation sequence

NPT: When applicable, how care providers were allocated to each trial group

Does your paper address CONSORT subitem 8a? *

Copy and paste relevant sections from the manuscript (include quotes in quotation marks "like this" to indicate direct quotes from your manuscript), or elaborate on this item by providing additional information not in the ms, or briefly explain why the item is not applicable/relevant for your study

A between-participants (FTF MTI vs computer-mediated MTI) repeated measures design was used. After assessing eligibility, young adults were randomly assigned using Excel's (Microsoft Corp) random function to participate in either an FTF MTI (n=75) or a computer-mediated MTI (n=75) at Time 1 (Figure 1). Both types of interviews were guided by the identical interview script. Of these, 3 participants who were originally assigned to the computer-mediated condition completed FTF interviews due to a computer software problem (the interviewer's prompt did not appear on the participants' screen and thus, these interviews were completed FTF).

8b) Type of randomisation; details of any restriction (such as blocking and block size)

Does your paper address CONSORT subitem 8b? *

Copy and paste relevant sections from the manuscript (include quotes in quotation marks "like this" to indicate direct quotes from your manuscript), or elaborate on this item by providing additional information not in the ms, or briefly explain why the item is not applicable/relevant for your study

A between-participants (FTF MTI vs computer-mediated MTI) repeated measures design was used. After assessing eligibility, young adults were randomly assigned using Excel's (Microsoft Corp) random function to participate in either an FTF MTI (n=75) or a computer-mediated MTI (n=75) at Time 1 (Figure 1). Both types of interviews were guided by the identical interview script. Of these, 3 participants who were originally assigned to the computer-mediated condition completed FTF interviews due to a computer software problem (the interviewer's prompt did not appear on the participants' screen and thus, these interviews were completed FTF).

9) Mechanism used to implement the random allocation sequence (such as sequentially numbered containers), describing any steps taken to conceal the sequence until interventions were assigned

Does your paper address CONSORT subitem 9? *

Copy and paste relevant sections from the manuscript (include quotes in quotation marks "like this" to indicate direct quotes from your manuscript), or elaborate on this item by providing additional information not in the ms, or briefly explain why the item is not applicable/relevant for your study

Interviewers were aware of the condition. A between-participants (FTF MTI vs computer-mediated MTI) repeated measures design was used. After assessing eligibility, young adults were randomly assigned using Excel's (Microsoft Corp) random function to participate in either an FTF MTI (n=75) or a computer-mediated MTI (n=75) at Time 1 (Figure 1). Both types of interviews were guided by the identical interview script. Of these, 3 participants who were originally assigned to the computer-mediated condition completed FTF interviews due to a computer software problem (the interviewer's prompt did not appear on the participants' screen and thus, these interviews were completed FTF).

10) Who generated the random allocation sequence, who enrolled participants, and who assigned participants to interventions

Does your paper address CONSORT subitem 10? *

Copy and paste relevant sections from the manuscript (include quotes in quotation marks "like this" to indicate direct quotes from your manuscript), or elaborate on this item by providing additional information not in the ms, or briefly explain why the item is not applicable/relevant for your study

KDL generated the random allocation sequence using excel random function, enrolled participants, and assigned participants to interventions.

11a) If done, who was blinded after assignment to interventions (for example, participants, care providers, those assessing outcomes) and how

NPT: Whether or not administering co-interventions were blinded to group assignment

11a-i) Specify who was blinded, and who wasn't

Specify who was blinded, and who wasn't. Usually, in web-based trials it is not possible to blind the participants [1, 3] (this should be clearly acknowledged), but it may be possible to blind outcome assessors, those doing data analysis or those administering co-interventions (if any).

	1	2	3	4	5	
subitem not at all important	○	○	○	○	◉	essential

Clear selection

Does your paper address subitem 11a-i? *

Copy and paste relevant sections from the manuscript (include quotes in quotation marks "like this" to indicate direct quotes from your manuscript), or elaborate on this item by providing additional information not in the ms, or briefly explain why the item is not applicable/relevant for your study

Interviewers were aware of condition, but participants were not. Coders were blind to what transcripts they were coding. FTF interviews were audio recorded, transcribed into text files, and proofread. Each interview was transcribed by 1 of the 5 undergraduate research assistants. To ensure confidentiality, identifying information that participants inadvertently provided during the interviews, such as the name of a participant's high school, was deleted. Transcribers also deleted any information from transcripts that could reveal the interview format (FTF or computer-mediated), such as the sounds "um," "ah," or "uh," which participants produced during FTF MTIs. Computer-mediated MTIs automatically produced written transcripts. To help mask the interview format (FTF or computer-mediated MTIs), the font size, and font type of all transcripts were reformatted to be identical by the same 5 undergraduate students. Typed utterances (eg, uh, um) and symbols (eg, emojis) were also deleted from transcripts to prevent revealing the interview format. Transcribers were unaware of the study's hypotheses but were aware that 2 types of interviews were conducted (ie, FTF vs computer-mediated MTIs).

11a-ii) Discuss e.g., whether participants knew which intervention was the "intervention of interest" and which one was the "comparator"

Informed consent procedures (4a-ii) can create biases and certain expectations - discuss e.g., whether participants knew which intervention was the "intervention of interest" and which one was the "comparator".

	1	2	3	4	5	
subitem not at all important	○	○	○	○	◉	essential

Clear selection

Does your paper address subitem 11a-ii?

Copy and paste relevant sections from the manuscript (include quotes in quotation marks "like this" to indicate direct quotes from your manuscript), or elaborate on this item by providing additional information not in the ms, or briefly explain why the item is not applicable/relevant for your study

Interviewers were aware of condition, but participants were not.

11b) If relevant, description of the similarity of interventions

(this item is usually not relevant for ehealth trials as it refers to similarity of a placebo or sham intervention to a active medication/intervention)

Does your paper address CONSORT subitem 11b? *

Copy and paste relevant sections from the manuscript (include quotes in quotation marks "like this" to indicate direct quotes from your manuscript), or elaborate on this item by providing additional information not in the ms, or briefly explain why the item is not applicable/relevant for your study

Computer-mediated interviews were conducted using the identical 4-page script that guided the FTF MTIs. Computer-mediated MTIs were completed via computer, with the interviewer and participant located in adjacent rooms within the same research suite used for the FTF MTIs. However, the interviewer and interviewee never met in person. Upon arrival, a research assistant greeted participants, administered Time 1 assessments, and provided instructions for using the computer's instant messaging software to communicate during the motivational-type interview. LAN Instant Messenger software (version 1.2.35, Qualia Digital Solutions) was used to conduct computer-mediated interviews and computer-mediated MTIs were saved as text files.

12a) Statistical methods used to compare groups for primary and secondary outcomes

NPT: When applicable, details of whether and how the clustering by care providers or centers was addressed

Does your paper address CONSORT subitem 12a? *

Copy and paste relevant sections from the manuscript (include quotes in quotation marks "like this" to indicate direct quotes from your manuscript), or elaborate on this item by providing additional information not in the ms, or briefly explain why the item is not applicable/relevant for your study

Due to nonnormality violations, Mann-Whitney nonparametric tests were used to compare sustain talk and change talk between the two experimental conditions. Negative binomial regression models were used to predict marijuana use.

12a-i) Imputation techniques to deal with attrition / missing values

Imputation techniques to deal with attrition / missing values: Not all participants will use the intervention/comparator as intended and attrition is typically high in ehealth trials. Specify how participants who did not use the application or dropped out from the trial were treated in the statistical analysis (a complete case analysis is strongly discouraged, and simple imputation techniques such as LOCF may also be problematic [4]).

	1	2	3	4	5	
subitem not at all important	○	○	○	○	◉	essential

Clear selection

Does your paper address subitem 12a-i? *

Copy and paste relevant sections from the manuscript (include quotes in quotation marks "like this" to indicate direct quotes from your manuscript), or elaborate on this item by providing additional information not in the ms, or briefly explain why the item is not applicable/relevant for your study

CONSORT (Consolidated Standards of Reporting Trials) diagram showing the flow of participants through Time 1 (baseline assessment) and Time 2 (follow-up assessment).

12b) Methods for additional analyses, such as subgroup analyses and adjusted analyses

Does your paper address CONSORT subitem 12b? *

Copy and paste relevant sections from the manuscript (include quotes in quotation marks "like this" to indicate direct quotes from your manuscript), or elaborate on this item by providing additional information not in the ms, or briefly explain why the item is not applicable/relevant for your study

Your answer

X26) REB/IRB Approval and Ethical Considerations [recommended as subheading under "Methods"] (not a CONSORT item)

X26-i) Comment on ethics committee approval

	1	2	3	4	5	
subitem not at all important	○	○	○	○	◉	essential

Clear selection

Does your paper address subitem X26-i?

Copy and paste relevant sections from the manuscript (include quotes in quotation marks "like this" to indicate direct quotes from your manuscript), or elaborate on this item by providing additional information not in the ms, or briefly explain why the item is not applicable/relevant for your study

This study was reviewed and approved by the University of Texas at El Paso Institutional Review Board (approval number 219115-2). All participants read and provided informed consent before proceeding with the survey and random assignment to an experimental condition. Participants were assured of confidentiality and created a self-generated ID number to ensure the anonymity of responses while permitting subsequent pairing of Time 1 (baseline) and Time 2 (follow-up) assessments.

x26-ii) Outline informed consent procedures

Outline informed consent procedures e.g., if consent was obtained offline or online (how? Checkbox, etc.?), and what information was provided (see 4a-ii). See [6] for some items to be included in informed consent documents.

	1	2	3	4	5	
subitem not at all important	○	○	○	○	◉	essential

Clear selection

Does your paper address subitem X26-ii?

Copy and paste relevant sections from the manuscript (include quotes in quotation marks "like this" to indicate direct quotes from your manuscript), or elaborate on this item by providing additional information not in the ms, or briefly explain why the item is not applicable/relevant for your study

This study was reviewed and approved by the University of Texas at El Paso Institutional Review Board (approval number 219115-2). All participants read and provided informed consent before proceeding with the survey and random assignment to an experimental condition. Participants were assured of confidentiality and created a self-generated ID number to ensure the anonymity of responses while permitting subsequent pairing of Time 1 (baseline) and Time 2 (follow-up) assessments.

X26-iii) Safety and security procedures

Safety and security procedures, incl. privacy considerations, and any steps taken to reduce the likelihood or detection of harm (e.g., education and training, availability of a hotline)

	1	2	3	4	5	
subitem not at all important	○	○	○	○	◉	essential

Clear selection

Does your paper address subitem X26-iii?

Copy and paste relevant sections from the manuscript (include quotes in quotation marks "like this" to indicate direct quotes from your manuscript), or elaborate on this item by providing additional information not in the ms, or briefly explain why the item is not applicable/relevant for your study

Participants were debrief about the purpose of the study and provided with local resources.

RESULTS

13a) For each group, the numbers of participants who were randomly assigned, received intended treatment, and were analysed for the primary outcome

NPT: The number of care providers or centers performing the intervention in each group and the number of patients treated by each care provider in each center

Does your paper address CONSORT subitem 13a? *

Copy and paste relevant sections from the manuscript (include quotes in quotation marks "like this" to indicate direct quotes from your manuscript), or elaborate on this item by providing additional information not in the ms, or briefly explain why the item is not applicable/relevant for your study

CONSORT (Consolidated Standards of Reporting Trials) diagram showing the flow of participants through Time 1 (baseline assessment) and Time 2 (follow-up assessment)

13b) For each group, losses and exclusions after randomisation, together with reasons

Does your paper address CONSORT subitem 13b? (NOTE: Preferably, this is shown in a CONSORT flow diagram) *

Copy and paste relevant sections from the manuscript (include quotes in quotation marks "like this" to indicate direct quotes from your manuscript), or elaborate on this item by providing additional information not in the ms, or briefly explain why the item is not applicable/relevant for your study

CONSORT (Consolidated Standards of Reporting Trials) diagram showing the flow of participants through Time 1 (baseline assessment) and Time 2 (follow-up assessment)

13b-i) Attrition diagram

Strongly recommended: An attrition diagram (e.g., proportion of participants still logging in or using the intervention/comparator in each group plotted over time, similar to a survival curve) or other figures or tables demonstrating usage/dose/engagement.

	1	2	3	4	5	
subitem not at all important	(selected)	○	○	○	○	essential

Clear selection

Does your paper address subitem 13b-i?

Copy and paste relevant sections from the manuscript or cite the figure number if applicable (include quotes in quotation marks "like this" to indicate direct quotes from your manuscript), or elaborate on this item by providing additional information not in the ms, or briefly explain why the item is not applicable/relevant for your study

Your answer

14a) Dates defining the periods of recruitment and follow-up

Does your paper address CONSORT subitem 14a? *

Copy and paste relevant sections from the manuscript (include quotes in quotation marks "like this" to indicate direct quotes from your manuscript), or elaborate on this item by providing additional information not in the ms, or briefly explain why the item is not applicable/relevant for your study

Questionnaires administered at Time 1 (baseline assessment: May 2015-October 2015) and Time 2 (follow-up assessment: July 2015-December 2015) assessed the frequency of marijuana use during the 2-month period preceding each assessment.

14a-i) Indicate if critical “secular events” fell into the study period

Indicate if critical “secular events” fell into the study period, e.g., significant changes in Internet resources available or “changes in computer hardware or Internet delivery resources”

	1	2	3	4	5	
subitem not at all important	○	○	○	◉	○	essential

Clear selection

Does your paper address subitem 14a-i?

Copy and paste relevant sections from the manuscript (include quotes in quotation marks "like this" to indicate direct quotes from your manuscript), or elaborate on this item by providing additional information not in the ms, or briefly explain why the item is not applicable/relevant for your study

We are not aware of any secular events.

14b) Why the trial ended or was stopped (early)

Does your paper address CONSORT subitem 14b? *

Copy and paste relevant sections from the manuscript (include quotes in quotation marks "like this" to indicate direct quotes from your manuscript), or elaborate on this item by providing additional information not in the ms, or briefly explain why the item is not applicable/relevant for your study

Participants were told they could stop or not answer questions during the interview.

15) A table showing baseline demographic and clinical characteristics for each group

NPT: When applicable, a description of care providers (case volume, qualification, expertise, etc.) and centers (volume) in each group

Does your paper address CONSORT subitem 15? *

Copy and paste relevant sections from the manuscript (include quotes in quotation marks "like this" to indicate direct quotes from your manuscript), or elaborate on this item by providing additional information not in the ms, or briefly explain why the item is not applicable/relevant for your study

Your answer

15-i) Report demographics associated with digital divide issues

In ehealth trials it is particularly important to report demographics associated with digital divide issues, such as age, education, gender, social-economic status, computer/Internet/ehealth literacy of the participants, if known.

	1	2	3	4	5	
subitem not at all important	○	○	○	○	◉	essential

Clear selection

Does your paper address subitem 15-i? *

Copy and paste relevant sections from the manuscript (include quotes in quotation marks "like this" to indicate direct quotes from your manuscript), or elaborate on this item by providing additional information not in the ms, or briefly explain why the item is not applicable/relevant for your study

To be eligible for the study, young adults had to express ambivalence about their level of marijuana use, as determined by responses to several items on the eligibility survey described below in the Eligibility Assessment section. Young adults were recruited from a large urban university in the Southwestern United States. Recruitment flyers were posted at several locations throughout the university campus.

16) For each group, number of participants (denominator) included in each analysis and whether the analysis was by original assigned groups

16-i) Report multiple “denominators” and provide definitions

Report multiple “denominators” and provide definitions: Report N’s (and effect sizes) “across a range of study participation [and use] thresholds” [1], e.g., N exposed, N consented, N used more than x times, N used more than y weeks, N participants “used” the intervention/comparator at specific pre-defined time points of interest (in absolute and relative numbers per group). Always clearly define “use” of the intervention.

	1	2	3	4	5	
subitem not at all important	○	○	○	○	◉	essential

Clear selection

Does your paper address subitem 16-i? *

Copy and paste relevant sections from the manuscript (include quotes in quotation marks "like this" to indicate direct quotes from your manuscript), or elaborate on this item by providing additional information not in the ms, or briefly explain why the item is not applicable/relevant for your study

Table 1 and 2 includes a statement for number of participants. Five motivational-type interview transcripts were lost during the transcription process. Table 3 mentions "The model includes 106 participants with valid observations for all variables in the model".

16-ii) Primary analysis should be intent-to-treat

Primary analysis should be intent-to-treat, secondary analyses could include comparing only "users", with the appropriate caveats that this is no longer a randomized sample (see 18-i).

	1	2	3	4	5	
subitem not at all important	●	○	○	○	○	essential

Clear selection

Does your paper address subitem 16-ii?

Copy and paste relevant sections from the manuscript (include quotes in quotation marks "like this" to indicate direct quotes from your manuscript), or elaborate on this item by providing additional information not in the ms, or briefly explain why the item is not applicable/relevant for your study

We did not conduct intent-to-treat analyses.

17a) For each primary and secondary outcome, results for each group, and the estimated effect size and its precision (such as 95% confidence interval)

Does your paper address CONSORT subitem 17a? *

Copy and paste relevant sections from the manuscript (include quotes in quotation marks "like this" to indicate direct quotes from your manuscript), or elaborate on this item by providing additional information not in the ms, or briefly explain why the item is not applicable/relevant for your study

We provide effect sizes and 95% CI for our main outcome predicting marijuana use at the 2 month follow-up.

17a-i) Presentation of process outcomes such as metrics of use and intensity of use

In addition to primary/secondary (clinical) outcomes, the presentation of process outcomes such as metrics of use and intensity of use (dose, exposure) and their operational definitions is critical. This does not only refer to metrics of attrition (13-b) (often a binary variable), but also to more continuous exposure metrics such as “average session length”. These must be accompanied by a technical description how a metric like a “session” is defined (e.g., timeout after idle time) [1] (report under item 6a).

	1	2	3	4	5	
subitem not at all important	(●)	( )	( )	( )	( )	essential

Clear selection

Does your paper address subitem 17a-i?

Copy and paste relevant sections from the manuscript (include quotes in quotation marks "like this" to indicate direct quotes from your manuscript), or elaborate on this item by providing additional information not in the ms, or briefly explain why the item is not applicable/relevant for your study

We conducted motivational type interviews at baseline.

17b) For binary outcomes, presentation of both absolute and relative effect sizes is recommended

Does your paper address CONSORT subitem 17b? *

Copy and paste relevant sections from the manuscript (include quotes in quotation marks "like this" to indicate direct quotes from your manuscript), or elaborate on this item by providing additional information not in the ms, or briefly explain why the item is not applicable/relevant for your study

We are not reporting binary outcomes. Our primary outcome is a count variable.

18) Results of any other analyses performed, including subgroup analyses and adjusted analyses, distinguishing pre-specified from exploratory

Does your paper address CONSORT subitem 18? *

Copy and paste relevant sections from the manuscript (include quotes in quotation marks "like this" to indicate direct quotes from your manuscript), or elaborate on this item by providing additional information not in the ms, or briefly explain why the item is not applicable/relevant for your study

A second set of analyses was conducted excluding nonusers and rerunning the negative binomial model predicting marijuana use at the 2-month follow-up. After excluding nonusers, we entered gender, experimental condition, the strength of commitment language, and the interaction between commitment language and condition. We found a similar pattern to the overall sample (Table 3) and the analyses that exclude nonusers of marijuana. The interaction between experimental conditions and the strength of Time 1 commitment language did predict Time 2 marijuana use (B=0.38, P=.02). We probed the interaction using negative binomial regression with a log link function to model the relationship between Time 1 commitment language and Time 2 marijuana use for each of the experimental conditions. For participants in the FTF MTI condition, Time 2 marijuana use decreased as the strength of Time 1 commitment language increased. However, this finding was not significant (B=–0.15, P=.20). For participants in the computer-mediated MTI condition, Time 2 marijuana use increased as the strength of Time 1 commitment language increased. However, this finding was not significant (B=0.14, P=.29).

18-i) Subgroup analysis of comparing only users

A subgroup analysis of comparing only users is not uncommon in ehealth trials, but if done, it must be stressed that this is a self-selected sample and no longer an unbiased sample from a randomized trial (see 16-iii).

	1	2	3	4	5	
subitem not at all important	◉	○	○	○	○	essential

Clear selection

Does your paper address subitem 18-i?

Copy and paste relevant sections from the manuscript (include quotes in quotation marks "like this" to indicate direct quotes from your manuscript), or elaborate on this item by providing additional information not in the ms, or briefly explain why the item is not applicable/relevant for your study

Your answer

19) All important harms or unintended effects in each group

(for specific guidance see CONSORT for harms)

Does your paper address CONSORT subitem 19? *

Copy and paste relevant sections from the manuscript (include quotes in quotation marks "like this" to indicate direct quotes from your manuscript), or elaborate on this item by providing additional information not in the ms, or briefly explain why the item is not applicable/relevant for your study

Your answer

19-i) Include privacy breaches, technical problems

Include privacy breaches, technical problems. This does not only include physical “harm” to participants, but also incidents such as perceived or real privacy breaches [1], technical problems, and other unexpected/unintended incidents. “Unintended effects” also includes unintended positive effects [2].

	1	2	3	4	5	
subitem not at all important	○	○	○	○	◉	essential

Clear selection

Does your paper address subitem 19-i?

Copy and paste relevant sections from the manuscript (include quotes in quotation marks "like this" to indicate direct quotes from your manuscript), or elaborate on this item by providing additional information not in the ms, or briefly explain why the item is not applicable/relevant for your study

Your answer

19-ii) Include qualitative feedback from participants or observations from staff/researchers

Include qualitative feedback from participants or observations from staff/researchers, if available, on strengths and shortcomings of the application, especially if they point to unintended/unexpected effects or uses. This includes (if available) reasons for why people did or did not use the application as intended by the developers.

	1	2	3	4	5	
subitem not at all important	(●)	( )	( )	( )	( )	essential

Clear selection

Does your paper address subitem 19-ii?

Copy and paste relevant sections from the manuscript (include quotes in quotation marks "like this" to indicate direct quotes from your manuscript), or elaborate on this item by providing additional information not in the ms, or briefly explain why the item is not applicable/relevant for your study

We did not assess

DISCUSSION

22) Interpretation consistent with results, balancing benefits and harms, and considering other relevant evidence

NPT: In addition, take into account the choice of the comparator, lack of or partial blinding, and unequal expertise of care providers or centers in each group

22-i) Restate study questions and summarize the answers suggested by the data, starting with primary outcomes and process outcomes (use)

Restate study questions and summarize the answers suggested by the data, starting with primary outcomes and process outcomes (use).

	1	2	3	4	5	
subitem not at all important	○	○	○	○	◉	essential

Clear selection

Does your paper address subitem 22-i? *

Copy and paste relevant sections from the manuscript (include quotes in quotation marks "like this" to indicate direct quotes from your manuscript), or elaborate on this item by providing additional information not in the ms, or briefly explain why the item is not applicable/relevant for your study

Your answer

22-ii) Highlight unanswered new questions, suggest future research

Highlight unanswered new questions, suggest future research.

	1	2	3	4	5	
subitem not at all important	○	○	○	○	◉	essential

Clear selection

Does your paper address subitem 22-ii?

Copy and paste relevant sections from the manuscript (include quotes in quotation marks "like this" to indicate direct quotes from your manuscript), or elaborate on this item by providing additional information not in the ms, or briefly explain why the item is not applicable/relevant for your study

Your answer

20) Trial limitations, addressing sources of potential bias, imprecision, and, if relevant, multiplicity of analyses

20-i) Typical limitations in ehealth trials

Typical limitations in ehealth trials: Participants in ehealth trials are rarely blinded. Ehealth trials often look at a multiplicity of outcomes, increasing risk for a Type I error. Discuss biases due to non-use of the intervention/usability issues, biases through informed consent procedures, unexpected events.

	1	2	3	4	5	
subitem not at all important	○	○	○	○	◉	essential

Clear selection

Does your paper address subitem 20-i? *

Copy and paste relevant sections from the manuscript (include quotes in quotation marks "like this" to indicate direct quotes from your manuscript), or elaborate on this item by providing additional information not in the ms, or briefly explain why the item is not applicable/relevant for your study

Your answer

21) Generalisability (external validity, applicability) of the trial findings

NPT: External validity of the trial findings according to the intervention, comparators, patients, and care providers or centers involved in the trial

21-i) Generalizability to other populations

Generalizability to other populations: In particular, discuss generalizability to a general Internet population, outside of a RCT setting, and general patient population, including applicability of the study results for other organizations

	1	2	3	4	5	
subitem not at all important	○	○	○	○	◉	essential

Clear selection

Does your paper address subitem 21-i?

Copy and paste relevant sections from the manuscript (include quotes in quotation marks "like this" to indicate direct quotes from your manuscript), or elaborate on this item by providing additional information not in the ms, or briefly explain why the item is not applicable/relevant for your study

Your answer

21-ii) Discuss if there were elements in the RCT that would be different in a routine application setting

Discuss if there were elements in the RCT that would be different in a routine application setting (e.g., prompts/reminders, more human involvement, training sessions or other co-interventions) and what impact the omission of these elements could have on use, adoption, or outcomes if the intervention is applied outside of a RCT setting.

	1	2	3	4	5	
subitem not at all important	○	○	○	○	◉	essential

Clear selection

Does your paper address subitem 21-ii?

Copy and paste relevant sections from the manuscript (include quotes in quotation marks "like this" to indicate direct quotes from your manuscript), or elaborate on this item by providing additional information not in the ms, or briefly explain why the item is not applicable/relevant for your study

Your answer

OTHER INFORMATION

23) Registration number and name of trial registry

Does your paper address CONSORT subitem 23? *

Copy and paste relevant sections from the manuscript (include quotes in quotation marks "like this" to indicate direct quotes from your manuscript), or elaborate on this item by providing additional information not in the ms, or briefly explain why the item is not applicable/relevant for your study

Unfortunately, no. We did not preregister the study.

24) Where the full trial protocol can be accessed, if available

Does your paper address CONSORT subitem 24? *

Cite a Multimedia Appendix, other reference, or copy and paste relevant sections from the manuscript (include quotes in quotation marks "like this" to indicate direct quotes from your manuscript), or elaborate on this item by providing additional information not in the ms, or briefly explain why the item is not applicable/relevant for your study

Your answer

25) Sources of funding and other support (such as supply of drugs), role of funders

Does your paper address CONSORT subitem 25? *

Copy and paste relevant sections from the manuscript (include quotes in quotation marks "like this" to indicate direct quotes from your manuscript), or elaborate on this item by providing additional information not in the ms, or briefly explain why the item is not applicable/relevant for your study

Your answer

X27) Conflicts of Interest (not a CONSORT item)

X27-i) State the relation of the study team towards the system being evaluated

In addition to the usual declaration of interests (financial or otherwise), also state the relation of the study team towards the system being evaluated, i.e., state if the authors/evaluators are distinct from or identical with the developers/sponsors of the intervention.

	1	2	3	4	5	
subitem not at all important	○	○	○	○	◉	essential

Clear selection

Does your paper address subitem X27-i?

Copy and paste relevant sections from the manuscript (include quotes in quotation marks "like this" to indicate direct quotes from your manuscript), or elaborate on this item by providing additional information not in the ms, or briefly explain why the item is not applicable/relevant for your study

Your answer

About the CONSORT EHEALTH checklist

As a result of using this checklist, did you make changes in your manuscript? *

- ○ yes, major changes
- ○ yes, minor changes
- ○ no

What were the most important changes you made as a result of using this checklist?

Your answer

How much time did you spend on going through the checklist INCLUDING making changes in your manuscript *

Your answer

As a result of using this checklist, do you think your manuscript has improved? *

- (x) yes
- ( ) no
- ( ) Other:

Would you like to become involved in the CONSORT EHEALTH group?

This would involve for example becoming involved in participating in a workshop and writing an "Explanation and Elaboration" document

- ( ) yes
- ( ) no
- ( ) Other:

Any other comments or questions on CONSORT EHEALTH

Your answer

STOP - Save this form as PDF before you click submit

To generate a record that you filled in this form, we recommend to generate a PDF of this page (on a Mac, simply select "print" and then select "print as PDF") before you submit it.

When you submit your (revised) paper to JMIR, please upload the PDF as supplementary file.

Don't worry if some text in the textboxes is cut off, as we still have the complete information in our database. Thank you!

Final step: Click submit !

Click submit so we have your answers in our database!

Submit

Clear form

Never submit passwords through Google Forms.

This content is neither created nor endorsed by Google. - Terms of Service - Privacy Policy

Does this form look suspicious? Report

Google Forms
